# Comprehensive Analysis of SWI/SNF Inactivation in Lung Adenocarcinoma Cell Models

**DOI:** 10.3390/cancers12123712

**Published:** 2020-12-10

**Authors:** Paola Peinado, Alvaro Andrades, Marta Cuadros, Maria Isabel Rodriguez, Isabel F. Coira, Daniel J. Garcia, Juan Carlos Álvarez-Perez, Carlos Baliñas-Gavira, Alberto M. Arenas, Juan Rodrigo Patiño-Mercau, Juan Sanjuan-Hidalgo, Octavio A. Romero, Luis M. Montuenga, Julian Carretero, Montserrat Sanchez-Cespedes, Pedro P. Medina

**Affiliations:** 1 Department of Biochemistry and Molecular Biology I, University of Granada, 18071 Granada, Spain; paola.peinado@genyo.es (P.P.); alande@ugr.es (A.A.); carlosalvarez@ugr.es (J.C.Á.-P.); carlos.balinas@genyo.es (C.B.-G.); amam@ugr.es (A.M.A.); juanmercau@ugr.es (J.R.P.-M.); 2 GENYO, Centre for Genomics and Oncological Research, Pfizer/University of Granada/Andalusian Regional Government, 18016 Granada, Spain; mcuadros@ugr.es (M.C.); maria.rodriguez@genyo.es (M.I.R.); djgargar@ugr.es (D.J.G.); juan.sanjuan@genyo.es (J.S.-H.); 3 Health Research Institute of Granada (ibs.Granada), 18014 Granada, Spain; 4 Department of Biochemistry and Molecular Biology III and Immunology, University of Granada, 18016 Granada, Spain; 5 School of Pharmaceutical Sciences, University of Geneva, 1211 Geneva, Switzerland; isabel.fernandezcoira@unige.ch; 6 Institute of Pharmaceutical Sciences of Western Switzerland, University of Geneva, 1211 Geneva, Switzerland; 7 Genes and Cancer Group, Cancer Epigenetics and Biology Program (PEBC), Bellvitge Biomedical Research Institute (IDIBELL), Hospitalet de Llobregat, 08908 Barcelona, Spain; oromero@carrerasresearch.org (O.A.R.); mscespedes@carrerasresearch.org (M.S.-C.); 8 Solid Tumors Program, Centro de Investigación Médica Aplicada and Department of Pathology, Anatomy and Physiology, University of Navarra Pamplona, 31009 Pamplona, Spain; lmontuenga@unav.es; 9 Navarra’s Health Research Institute (IDISNA) and CIBERONC, 31008 Pamplona, Spain; 10 Department of Physiology, University of Valencia, 46100 Burjassot, Valencia, Spain; julian.carretero@uv.es

**Keywords:** SWI/SNF complex, lung cancer, lung adenocarcinoma, epigenetics, cell models, multi-omics

## Abstract

**Simple Summary:**

Mammalian SWI/SNF complexes regulate gene expression by reorganizing the way DNA is packaged into chromatin. SWI/SNF subunits are recurrently altered in tumors at multiple levels, including DNA mutations as well as alteration of the levels of RNA and protein. Cancer cell lines are often used to study SWI/SNF function, but their patterns of SWI/SNF alterations can be complex. Here, we present a comprehensive characterization of DNA mutations and RNA and protein expression of SWI/SNF members in 38 lung adenocarcinoma (LUAD) cell lines. We show that over 85% of our cell lines harbored at least one alteration in one SWI/SNF subunit. In addition, over 75% of our cell lines lacked expression of at least one SWI/SNF subunit at the protein level. Our catalog will help researchers choose an appropriate cell line model to study SWI/SNF function in LUAD.

**Abstract:**

Mammalian SWI/SNF (SWitch/Sucrose Non-Fermentable) complexes are ATP-dependent chromatin remodelers whose subunits have emerged among the most frequently mutated genes in cancer. Studying SWI/SNF function in cancer cell line models has unveiled vulnerabilities in SWI/SNF-mutant tumors that can lead to the discovery of new therapeutic drugs. However, choosing an appropriate cancer cell line model for SWI/SNF functional studies can be challenging because SWI/SNF subunits are frequently altered in cancer by various mechanisms, including genetic alterations and post-transcriptional mechanisms. In this work, we combined genomic, transcriptomic, and proteomic approaches to study the mutational status and the expression levels of the SWI/SNF subunits in a panel of 38 lung adenocarcinoma (LUAD) cell lines. We found that the SWI/SNF complex was mutated in more than 76% of our LUAD cell lines and there was a high variability in the expression of the different SWI/SNF subunits. These results underline the importance of the SWI/SNF complex as a tumor suppressor in LUAD and the difficulties in defining altered and unaltered cell models for the SWI/SNF complex. These findings will assist researchers in choosing the most suitable cellular models for their studies of SWI/SNF to bring all of its potential to the development of novel therapeutic applications.

## 1. Introduction

Advances in DNA sequencing technologies have helped in deciphering the mutational landscape of human cancers. However, the full picture of alterations in human cancers is far from complete unless we integrate multi-omics data. In vitro models are extensively used in bench research because they allow researchers to test new hypotheses in simple but clinically translatable experiments. In this way, in vitro models are often the first step for studies that aim to improve the diagnosis, the prognosis, or the treatment of diseases. However, these models must be comprehensively characterized at the molecular level for researchers to be able to choose the most appropriate model for their study and interpret the results of their experiments [1,2].

Understanding the characteristics of in vitro models is especially important when studying multiprotein complexes whose arrangement determines their functionality in tumor contexts. In this study, we focus on the multiprotein complex SWI/SNF (SWitch/Sucrose Non-Fermentable), which is an ATP-dependent chromatin-remodeling complex that controls nucleosome positioning and recruits other chromatin binding factors. This complex has been considered as the most mutated chromatin regulator and one of the most mutated tumor suppressors with alterations found in almost 20% of all human neoplasias [3,4]. For this reason, many researchers are studying this complex and looking for new strategies to treat SWI/SNF-mutant tumors.

Currently, 29 SWI/SNF subunits have been described but only 10 to 15 of them coexist in a single complex [5]. Indeed, three different SWI/SNF complexes have been defined based on their subunit composition: canonical BAF (BRM/BRG1 Associated Factors), PBAF (polybromo-associated BAF complexes), and a recently discovered non-canonical BAF (ncBAF) [6,7,8]. The subunit arrangement of these complexes can have a functional impact for the cell. Moreover, when certain subunits harbor loss-of-function mutations, they can create vulnerabilities that can be targetable in the clinical practice (reviewed in [9]). However, this information has only been validated in certain tumor contexts and with a very limited number of subunits.

In the case of lung cancer, which is the deadliest type of malignancy worldwide [10], translational applications have only been developed against *SMARCA4*-mutant tumors [11,12,13,14,15,16,17]. Since the discovery of genetic alterations in *SMARCA4* in both lung primary tumors [18] and cell lines [19,20], this subunit has gained an increased interest and is considered the most mutated SWI/SNF subunit in lung adenocarcinoma (LUAD) [21,22]. However, subsequent studies have also found a remarkable percentage of mutations in other SWI/SNF subunits, such as *ARID1A* [22,23,24], that can contribute to its functional inactivation. Moreover, genetic alterations are not the only source of modifications of SWI/SNF activity: additional mechanisms of transcriptional and post-transcriptional regulation can contribute to SWI/SNF complex inactivation [25,26]. All these factors hinder the choice of the right cell line for developing functional models or studying targetable synthetic lethalities in clinical practice. Hence, this paper aims to build a solid catalog of LUAD cell lines where researchers can have an integrative overview of the most relevant subunits of the SWI/SNF complex to choose the most suitable cellular models for their study.

## 2. Results

### 2.1. More than 75% of LUAD Cell Lines Have a Mutated SWI/SNF Subunit

We selected a panel of representative LUAD cell lines that are commonly used (quantified by the number of Pubmed citations, see Figure S1A) and that combine different genetic and clinical backgrounds (see Figure S1B and Table S1). We analyzed the mutational status of 20 SWI/SNF subunits that were detected in a SMARCA4 immunoprecipitation in a non-tumor lung cell line (Peinado, P. et al. [27], Manuscript in preparation) and the top five LUAD driver genes identified by Bailey and colleagues [22] (see Table S2) using capture-based DNA sequencing.

**Figure 1:**
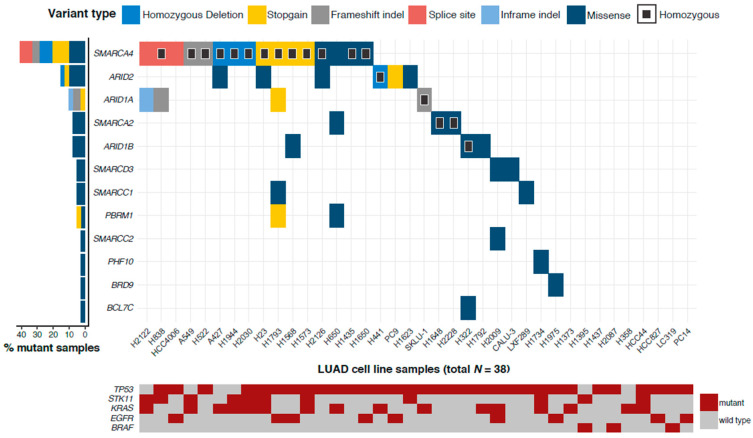

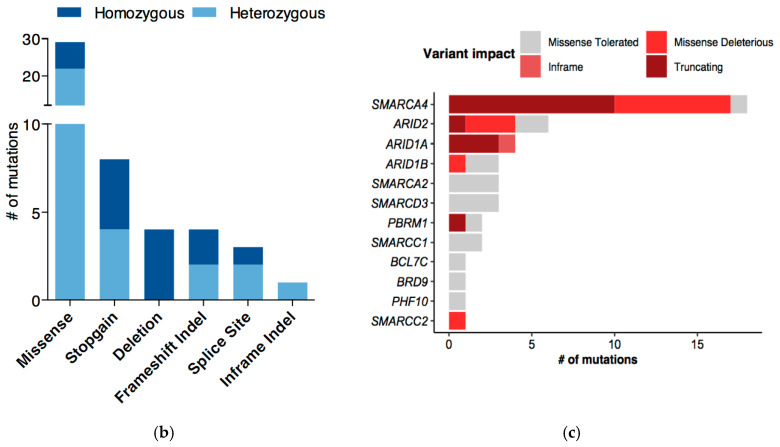
Mutational study of the SWI/SNF (SWitch/Sucrose Non-Fermentable) complex in lung adenocarcinoma (LUAD) cell lines. (**a**) Mutation profile of 20 SWI/SNF complex subunits in 38 LUAD cell lines. The Y axis represents all the subunits that had at least one genetic alteration in one LUAD cell line. Homozygous mutations are depicted with a black square. The *X* axis gathers all LUAD cell lines included in this study. On the left, mutation frequencies of these SWI/SNF subunits in LUAD cell lines. (**b**) Distribution of the different variant types found in SWI/SNF subunits in our 38 LUAD cell lines. Light blue shows those genetic alterations that were heterozygous. Dark blue depicts homozygous mutations. (**c**) Functional prediction of the mutations found in SWI/SNF subunits in our panel of 38 LUAD cell lines using SIFT.

Twenty-nine out of the 38 LUAD cell lines (76.3%) harbored at least one genetic alteration in SWI/SNF genes (Figure 1a). Specifically, 12 out of the 20 (60.0%) SWI/SNF subunits were mutated or had homozygous deletions in our panel of LUAD cell lines, accumulating 49 genetic alterations. Indeed, the top five most cited LUAD cell lines had a mutation affecting a lung SWI/SNF subunit. *SMARCA4* was the top mutated SWI/SNF gene (mutation rate = 42.1%) as seen in previous studies [13,15]. *ARID2* (15.8%), *ARID1A* (10.5%), *ARID1B* (7.9%), and *SMARCA2* (7.9%) were also part of the top five most mutated SWI/SNF subunit genes.

To corroborate our observations with external data, we compared our results with those reported by the Cancer Cell Line Encyclopedia [28]. In general, our mutational data highly agreed with the CCLE although we found some discrepancies that are explained in further detail in Notes and Figures S2–S5.

We also included an analysis of the mutational status of the top five LUAD driver genes in the same panel of cell lines (Figure 1a). On the one hand, we observed that, among these driver genes, *BRAF* was the only one that did not harbor any mutations when there was a mutated SWI/SNF subunit. On the other hand, due to the high mutation rate of *TP53* in our LUAD cell lines (84.2%), most SWI/SNF mutant cell lines were also *TP53*-mutant. Only 25% of *SMARCA4* and *ARID1A* mutant samples and 16% of *ARID2* mutant samples had a wild-type *TP53*. This information could be especially valuable when studying the influence of a particular genetic background on a mutant SWI/SNF complex and it could give rise to new therapeutic approaches. To evaluate whether any co-occurrence or mutual exclusion of mutations was statistically significant, we analyzed a larger cohort from TCGA-LUAD (*n* = 574). We considered all possible pairs between SWI/SNF subunits and the five tested LUAD drivers (see Figure S1C). SWI/SNF mutations significantly overlapped with *TP53* mutations in TCGA data (*p* = 0.0018). However, this overlap could not be corroborated in our cell line data (*p* = 0.61). Nevertheless, analysis of co-occurrence and mutual exclusion of mutations should be interpreted with caution because they are affected by multiple external variables and some significant results might be statistical artifacts [29].

Among all genetic alterations that affected SWI/SNF subunits, almost 60% were missense mutations (Figure 1b). For this reason, we predicted the functional impact of these missense mutations using the SIFT algorithm [30]. Based on SIFT predictions, we estimated that 41% of missense mutations could be “deleterious” and impact the functionality of the protein (Figure 1c).

### 2.2. mRNA Profiles of LUAD Cell Lines Show a Transcriptional De-Regulation of the SWI/SNF Complex

**Figure 2:**
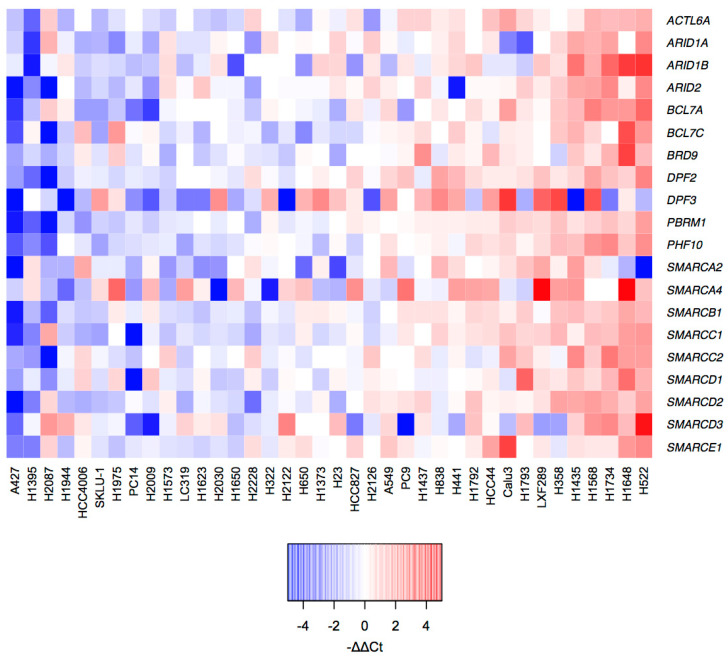
Transcriptional analysis of the SWI/SNF complex in LUAD cell lines. Heatmap of mRNA expression changes within the LUAD cell lines in 20 SWI/SNF subunits (−DDCt was calculated using the median DCt for each of the measured genes). The *Y* axis represents all measured SWI/SNF subunits. The *X* axis contains the 38 LUAD cell lines of our study.

The high mutation rate is not the only cause that can affect the function of the SWI/SNF complex. Other mechanisms, such as epigenetic silencing through promoter hypermethylation or post-transcriptional regulation, can lead to an alteration of SWI/SNF expression [25,26]. We analyzed the mRNA levels of the same 20 SWI/SNF subunits in our 38 LUAD cell lines by RT-qPCR. We evaluated the intrinsic variability of mRNA expression among cell lines using the median DCt for each gene as the normalization value to calculate a −DDCt (Figure 2 and Figure S6). With this representation, our panel of LUAD cell lines displayed two tendencies. On the one hand, there were cell lines with low relative expression of most of the SWI/SNF complex (e.g., A427 and H1395). On the other hand, our panel also contained cell lines with high relative mRNA levels of most SWI/SNF subunits (e.g., H522 and H1648).

**Figure 3:**
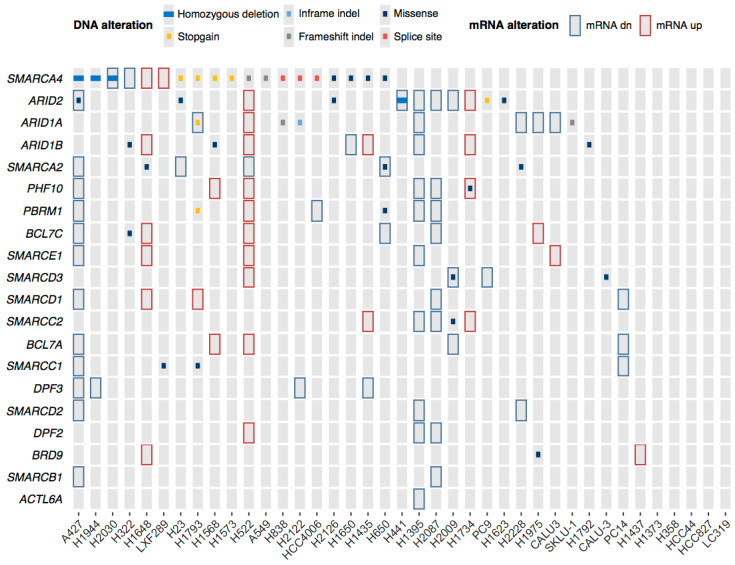
Summary of DNA and RNA alterations of the analyzed SWI/SNF subunits in our collection of LUAD cell lines. Copy number alterations, point mutations, short indels, and alterations of mRNA levels are represented. To find alterations in mRNA expression, robust Z scores were calculated by subtracting the median DCt for each gene across all cell lines from the DCt for each gene in each cell line, and then dividing by the median absolute deviation for each gene across all cell lines. The cutoffs for down- or up-regulation were robust Z < −2 or robust Z > 2, respectively.

To provide a complete resource of SWI/SNF alterations in our panel of 38 LUAD cell lines, we combined data of DNA and mRNA alterations (Figure 3). Intriguingly, some cell lines, such as H1395, had most SWI/SNF subunits downregulated despite lacking DNA alterations.

### 2.3. Genetic and Epigenetic Factors Contribute to the Protein Loss of the ATPases and ARID Subunits

**Figure 4:**
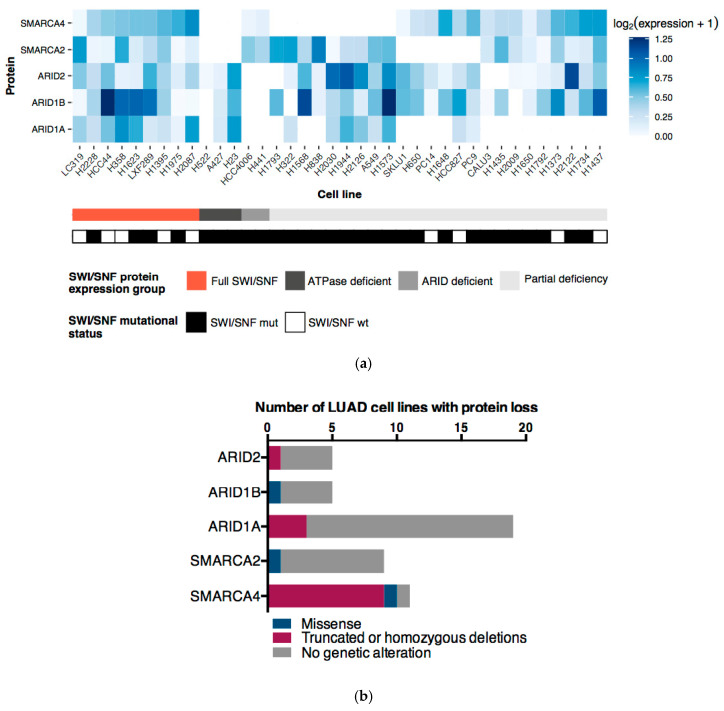
Protein expression profile of ATPases and ARID subunits of the SWI/SNF complex in LUAD cell lines. (**a**) Heatmap with normalized protein expression values of the ATPases and ARIDs subunits of the SWI/SNF in 38 LUAD cell lines. Zero values correspond to the absence of a band in the Western blot (see Figure S7). The first line below depicts the classification of LUAD cell lines based upon protein expression of the ATPases and ARIDs subunits. The second line shows the mutational status of the SWI/SNF complex considering all 20 subunits analyzed in this study (**b**) Causative analysis of the protein loss of the ATPases and ARID subunits in LUAD cell lines.

Generally, the ATPases (SMARCA4 and SMARCA2) and ARID subunits (ARID1A, ARID1B, and ARID2) are used to classify SWI/SNF complexes as BAF, PBAF, or ncBAF. We performed Western blot analysis for these subunits to evaluate which SWI/SNF complexes could be found in the 38 LUAD cell lines and in NL20, our control non-tumor lung cell line (Figure 4a). In contrast with NL20 (Figure S7), only 23.7% of the LUAD cell lines had detectable protein levels of the two ATPases and the three ARIDs. However, no LUAD cell line lacked all of the five analyzed proteins, supporting the idea that there may always be residual SWI/SNF complexes controlling gene expression (reviewed in [31]).

According to this Western Blot analysis, ARID1A was the most commonly lost subunit in LUAD cell lines, as we observed it in 19 out of 38 cell lines (50.0%). This was followed by the ATPase subunits SMARCA4 and SMARCA2, which were lost in 12 (31.6%) and nine (23.7%) LUAD cell lines, respectively. Among these three proteins, we observed different explanations for the protein loss (Figure 4b). On the one hand, 84% of the ARID1A loss and 89% of the SMARCA2 loss could not be explained by any genetic alterations. On the other hand, 83% of SMARCA4 losses were directly related to truncating mutations. Remarkably, most SMARCA4 mutations in cell lines were homozygous (Figure 1a), which could partially explain the strong correlation between its mutation and lack of protein. In general, these results also support our previous observation that there is a combination of genetic, epigenetic, and post-translational regulations that influence the expression and, therefore, the functionality of the SWI/SNF complex.

### 2.4. ATPases and ARID Protein Expression Profiles Define Four LUAD Cell Line Subgroups

The protein expression profiles of the five analyzed SWI/SNF subunits also allowed us to distinguish four subgroups of LUAD cell lines (Figure 4a). The first subgroup gathered all cell lines that showed detectable levels of all SWI/SNF proteins and it comprised nine cell lines (23.7%). Inside this subgroup, we could find five cell lines (LC319, HCC44, H358, H1395, and H2087) that were wild-type for all the 20 SWI/SNF subunits analyzed in this study. These cell lines could be a good reference of wild-type lung SWI/SNF contexts. Second, there was an ATPase deficient subgroup where we found three cell lines (7.9%). This subgroup has previously been observed in other studies [32,33,34] and these data restrict the widespread idea of SMARCA2/4 synthetic lethality proposed by Oike and colleagues to only certain genetic contexts [35]. Third, we defined a subgroup of ARID-deficient cell lines comprising H441 and HCC4006. This observation suggests that ARID1A/B synthetic lethality [36] is also limited to specific contexts. Moreover, this ARID-deficient subgroup could support the existence of the recently described ncBAF that lacks any ARID subunit [7,8]. To corroborate this observation, we analyzed the protein expression of BRD9, a specific ncBAF subunit (see Figure S8). Both ARID-deficient cell lines expressed BRD9 but their protein levels were lower than that observed in HCC44, one of the cell lines of the Full-SWI/SNF subgroup. Finally, we defined a fourth subgroup of LUAD cell lines bearing partial SWI/SNF loss in various combinations that reflect the diversity of assembly even within the same histological type of tumor. Overall, our observations emphasize the heterogeneity of the SWI/SNF status in LUAD cell lines, which must be taken into account when using these cell lines as in vitro *models* for studying the functionality of the SWI/SNF complex.

## 3. Discussion

Now that the importance of the SWI/SNF complex in cancer is more evident (reviewed in [37]), there is a need for well-designed studies to find new associations between the status of the SWI/SNF complex and clinical outcomes. Our study provides a resource for researchers where they can find a compilation of the most frequently used LUAD cell lines and their SWI/SNF profile at the genomic, transcriptomic, and protein levels of the five most characteristic and recurrently altered subunits.

Identifying what types of SWI/SNF subcomplexes are present in our in vitro models is key to developing representative functional studies. Indeed, a recent study has shown that the genomic distribution of the different SWI/SNF subcomplexes (BAF, PBAF, and ncBAF) differs among them and underlies a functional specificity of each of these subcomplexes and that could be important for developing certain targetable vulnerabilities [38].

In this work, we found that more than 76% of our panel of LUAD cell lines has a genetic alteration in at least one subunit of the SWI/SNF complex. Moreover, most of these genetic alterations may have an impact on the functionality of the mutated subunit, even those that were missense mutations, according to SIFT predictions. Additionally, we have observed some LUAD cell lines displaying either a transcriptional downregulation or upregulation of many SWI/SNF subunits, suggesting a complex regulatory layer of the SWI/SNF expression. Importantly, these types of alterations have also been described in patients [18,25], emphasizing the translational implications that studying solid cellular models could have in the clinic.

According to several studies, mutation or loss of expression of a SWI/SNF subunit does not fully inactivate the SWI/SNF complex but creates alternative residual complexes that can drive genome regulation of tumor cells [35,36,39,40,41]. These residual complexes are key in several drug discovery procedures, and new therapies have arisen after studying SWI/SNF-mutant complexes (reviewed in [9,42]).

In this LUAD panel, we can find cellular models for mutant contexts of the most altered SWI/SNF subunits in LUAD: SMARCA4, ARID2, and ARID1A with a mutation frequency of 42.1%, 15.8%, and 10.5%, respectively [22,23,24]. Our mutational analysis shows that ARID2 is a highly mutated SWI/SNF subunit surpassing the mutation frequency of ARID1A, which is the subunit that is the main mutated SWI/SNF subunit in other tumor types (reviewed in [43]). In the case of lung cancer, SMARCA4 is the SWI/SNF subunit that displays the highest number of deleterious mutations considering splice-site, stopgain variants, frameshift indels, and large deletions. Sixty-six percent (12/18) of the deleterious mutations found in the 20 SWI/SNF subunits of our study are found in SMARCA4. Furthermore, SMARCA4 concentrates 72% (13/18) of all of the homozygous alterations found in our analysis. It is not clear why SMARCA4 is the most deleteriously mutated SWI/SNF subunit in LUAD. Some studies have linked the proximity of the *SMARCA4* locus to the *STK11* locus (another well-known LUAD tumor suppressor gene) and the loss of heterozygosity that recurrently affects the short arm of the chromosome 19 in LUAD [44].

Our mutational analysis suggests the use of the A427, A549, H1568, H1573, H1793, H1944, H2030, H23, and H838 cell lines as reliable models of a defective SWI/SNF complex due to homozygous and deleterious mutations in *SMARCA4*. The cell lines H441 and SKLU-1 are two examples of a model with homozygous and deleterious mutations in *ARID2* and *ARID1A*, respectively. On the other hand, identifying a LUAD cell line without any aberrant SWI/SNF functionality is more difficult. Our RT-qPCR results and Western blot analysis suggest that alternative mechanisms to genetic alterations could also lead to an aberrant expression of the SWI/SNF subunits and damage the functionality of the complex. Intriguingly, several cell lines, such as H1395, display a transcriptional downregulation of most of the SWI/SNF subunits, which could be indicative of a still unknown regulatory system of the SWI/SNF complex subunits.

Currently, there is an increasing interest in using certain SWI/SNF-mutant models to develop new therapeutic strategies for cancer patients [9]. For example, it is known that SMARCA4 and ARID1A mutant tumors develop synthetic lethalities with other SWI/SNF subunits such as SMARCA2 and ARID1B, respectively [35,36]. However, so far, only SMARCA4-mutant tumors have been the target of synthetic lethal therapies in lung cancer [11,12,13,14,15,16,17]. Nevertheless, although SMARCA4 was the most mutated subunit, ARID1A was the SWI/SNF subunit with the highest incidence of protein loss in our 38 LUAD cell lines according to our Western blot analysis. This observation points out other interesting models that can be studied to discover new therapies. In addition, even though ARID1B is the most characteristic synthetic lethality described in ARID1A-mutant contexts, we found a subgroup of LUAD cell lines that not only lacked ARID1A and ARID1B, but also ARID2, the other possible ARID subunit. This subgroup of LUAD cell lines that we classified as “ARID-deficient” could be specially interesting for studying the role of the recently described non-canonical BAF complex (ncBAF) and its promising therapeutic vulnerabilities that have been already defined in other types of tumors [38].

Moreover, we found another subgroup of LUAD cell lines that we classified as “ATPase deficient” and that resembled a subgroup of LUAD patients with the loss of both SMARCA4 and SMARCA2 [32,45]. This subgroup has gained an additional interest since this specific profile leads to more aggressive outcomes [34]. For this reason, having these ATPase-deficient cellular models can facilitate the understanding of this specific condition and lay the foundation for the development of new therapeutic strategies to treat these aggressive LUAD tumors.

## 4. Materials and Methods

### 4.1. Lung Adenocarcinoma Cell Lines

Thirty-eight lung adenocarcinoma cell lines (A427, A549, CALU3, H1373, H1395, H1435, H1437, H1568, H1573, H1623, H1649, H1650, H1734, H1792, H1793, H1944, H1975, H2009, H2030, H2087, H2122, H2126, H2228, H23, H322, H358, H441, H522, H650, H838, HCC4006, HCC44, HCC827, LC319, LXF289, PC14, PC9, and SKLU1) were grown under standard conditions (37 °C, 5% carbon dioxide) in DMEM or RPMI 1640’s medium supplemented with glutamine, 10% fetal bovine serum, and 1% penicillin/streptomycin/amphotericin. Normal bronchial epithelial cells, NL20, were grown in Ham’s F12 medium with those supplements and culture conditions specified by the manufacturer.

### 4.2. DNA and RNA Extraction

Cell pellets were collected at 80% confluence. DNA and RNA were extracted from those pellets using an E.Z.N.A.^®^ Tissue DNA kit (Omega Bio-tek, Norcross, GA, USA) and TRI Reagent^®^ (Merck KGaA, Darmstadt, Germany), respectively.

### 4.3. Gene Capture and Targeted Sequencing

The baits for the gene capture against 20 SWI/SNF genes and the top five LUAD driver genes identified by Bailey and colleagues [22] were designed using NimbleDesign software (Roche, v4.0, Basel, Switzerland) (Table S2, Methods).

### 4.4. Deep Sequencing Data Analysis

The raw reads were aligned with the hg38 human genome using BWA-MEM. For the variant calling we combined bcftools with extensive filtering. In addition, we manually ‘rescued’ mutations from a non-paired Mutect2 analysis after thorough evaluation. Details on the pipelines, software versions, and external data sources are discussed in Methods.

### 4.5. In Silico Analysis of the SWI/SNF Complex in Lung Adenocarcinoma Cell Lines

We downloaded the merged mutation calls from the Cancer Cell Line Encyclopedia [28]. We converted the genomic coordinates of the mutations from hg19 to hg38 using liftOver in R (version 3.5.2, Bioconductor version 3.8,) [46].

### 4.6. Evaluation of the Functional Impact of Missense Mutations

To predict the functional impact of missense mutations, we used SIFT [30]. Only one consequence per variant allele was retained. We considered ‘possibly damaging’ mutations as ‘damaging’, and ‘possibly tolerated’ mutations as ‘tolerated’. If a variant originally annotated as ‘missense’ affected an isoform that was missing in the newer Ensembl 95, we assumed that it was ‘tolerated’.

### 4.7. Real-Time Quantitative Polymerase Chain Reaction

Total RNA (1 µg) was used to prepare cDNA with the RevertAid RT Kit (Thermo Scientific, Waltham, MA, USA). Real-time quantitative PCR (RT-qPCR) reactions followed KAPA SYBR^®^ FAST qPCR Master Mix (Merck KGaA, Darmstadt, Germany) recommendations. RT-qPCR was optimized using the Applied Biosystems 7900HT Real-Time PCR System (Thermo Scientific, Waltham, MA, USA). Relative expression was calculated using GAPDH as a reference gene and applying the DDCt method. Primers for each gene are shown in Table S3. All experiments were carried out in duplicate or triplicate.

### 4.8. Western Blot

Total protein was extracted from cells using cell lysis RIPA buffer and supplemented with a phosphatase and protease inhibitor cocktail; 60 µg of protein lysate was resolved by SDS–polyacrylamide gel electrophoresis (SDS-PAGE; 6%) and transferred to PVDF membranes, which were blocked with 5% non-fat milk in PBS-T and incubated overnight with the indicated antibody. The primary antibodies used are the following: anti-SMARCA4 (sc-17796, Santa Cruz Biotechnology, Dallas, TX, USA), SMARCA2 (D9E8B-XP, Cell Signaling Technology, Danvers, MA, USA), ARID1A (D2A8U, Cell Signaling Technology), ARID1B (ab57461, Abcam, Cambridge, UK), ARID2 (sc-166117, Santa Cruz Biotechnology), BRD9 (A303-781A, Bethyl Laboratories), and anti-BETA-ACTIN (A5441, Sigma Aldrich, Merck KGaA, Darmstadt, Germany). The membranes were then treated with HRP-conjugated anti-rabbit or anti-mouse secondary antibodies (P0448 and P0447, respectively, Dako, Agilent Technologies, Santa Clara, CA, USA). The target protein bands were visualized using Clarity Western ECL Substrate (BioRad, Hercules, CA, USA) and ImageQuant LAS4000 (GE Healthcare, Chicago, IL, USA). Protein bands were quantified using ImageJ software. We normalized the data with ACTB values and we transformed the data by applying the ‘log2 (normalized value +1)’.

### 4.9. TCGA Data Download

Harmonized mutational data from TCGA were downloaded from Genomic Data Commons [47] using the R package TCGAbiolinks. Mutations from the Mutect2 workflow were used.

### 4.10. Statistical Analysis

Statistical analyses were performed in R version 3.5.2 [48] or higher unless specified otherwise. Co-occurrence or mutual exclusion of mutations in gene pairs was analyzed by Fisher’s exact tests, and *p* values were adjusted for multiple testing using the Benjamini–Hochberg method.

### 4.11. Data availability

Cell line DNA sequencing data have been uploaded to the European Nucleotide Archive (ENA) under accession number PRJEB40655.

## 5. Conclusions

Overall, our study combines different approaches in an attempt to serve as a reference for investigators of the SWI/SNF complex and lung cancer. We emphasize the need for correct cellular models that resemble certain biological backgrounds more accurately. We have defined four subgroups of LUAD cell lines that share a specific signature regarding the SWI/SNF complex, which can help to address all the possible questions that are currently in the field.

## Supplementary Materials

The following are available online at https://www.mdpi.com/2072-6694/12/12/3712/s1, Figure S1: Bibliography and mutational profile of our LUAD cell line panel, Figure S2: De novo SWI/SNF complex mutations identified by our study in LUAD cell lines, Figure S3: SWI/SNF complex mutations with a difference in annotation between CCLE and our data, Figure S4: CCLE mutations in the lung SWI/SNF complex excluded from our analysis because of the effect on low complexity genomic regions, Figure S5: Lung SWI/SNF complex mutations exclusively described in CCLE, Figure S6: Transcriptional analysis of other SWI/SNF subunits. Figure S7: Western blot of SMARCA4, SMARCA2, ARID1A, ARID1B, and ARID2 in our 38 LUAD cell lines and a normal lung cell line (NL20). Figure S8: Western blot of BRD9 in the ARID-deficient cell lines and a WT SWI/SNF cell line. Table S1: Clinical features of our panel of LUAD cell lines, Table S2: DNA-Seq baits of the lung SWI/SNF subunits and the top five LUAD driver genes, Table S3: RT-qPCR oligonucleotides used.
