# Supplementary material for: Comprehensive Analysis of SWI/SNF Inactivation in Lung Adenocarcinoma Cell Models
Source: Cancers (Basel). 2020 Dec 10;12(12):3712. doi: 10.3390/cancers12123712 (PMC7763689; doi:10.3390/cancers12123712)
Supplement: Supplementary file 1 [file cancers-12-03712-s001.zip › cancers-1000530-supplementary/cancers-1000530-supplementary materials.docx]

Supplementary Materials: Comprehensive Analysis of SWI/SNF Inactivation in Lung Adenocarcinoma Cell Models

Paola Peinado, Alvaro Andrades, Marta Cuadros, Maria Isabel Rodriguez, Isabel F. Coira, Daniel J. Garcia, Juan Carlos Álvarez-Perez, Carlos Baliñas-Gavira, Alberto M. Arenas, Juan Rodrigo Patiño-Mercau, Juan Sanjuan-Hidalgo, Octavio A. Romero, Luis M. Montuenga, Julian Carretero, Montserrat Sanchez-Cespedes and Pedro P. Medina

1. Supplemental Methods

1.1. Gene Capture and Targeted Sequencing

300 ng of genomic DNA were fragmented using a Covaris S2 sonicator yielding 180–220 bp fragments. After end repair and adapter ligation, the adapter-ligated fragments were amplified by PCR (9 cycles). The PCR fragments were purified and the fragments with the correct size were selected. DNA was denatured and hybridized against biotinylated probes, which were then captured using streptavidin-bound magnetic beads. The DNA bound to the beads was isolated and amplified by PCR (14 cycles). The quality and the concentration of the DNA were evaluated using NanoDrop (Thermo Scientific) and BioAnalyzer (Agilent). The paired-end sequencing was performed on a NextSeq 500 instrument (Illumina) using a NextSeq 500/550 Mid Output Kit (Illumina), 2 × 150 cycles.

1.2. Deep Sequencing Data Analysis

The quality of the raw FASTQ sequencing files was evaluated using FastQC (v0.11.5, <http://www.bioinformatics.babraham.ac.uk/projects/fastqc>). Then, the adapter sequences were removed using Cutadapt [1] with the following options: -b AGATCGGAAGAGC -B AGATCGGAAGAGC –q 20 –m 50.

After trimming the adapters, the reads were aligned to the hg38 human genome (<http://hgdownload.cse.ucsc.edu/goldenPath/hg38/bigZips>) using BWA-MEM (v0.7.13-r1126) with the -M option. Afterwards, we used Picard (v2.1.1) to convert the SAM files to BAM format, sort the BAM files and mark PCR duplicates. Quality metrics were collected using Qualimap (v2.2.1) [2] and MultiQC (v1.7) [3].

We performed a non-paired mutational analysis combining two approaches. In our first approach, we used BCFtools applying the following filters: individual variant QUAL ≥ 20 and either total coverage ≥ 8 or ≥ 5 mutant reads. We also flagged as ‘LowFreq’ the mutations that had a mutant allele frequency below 20%:

“bcftools mpileup -f hg38.fa -R primary_targets.bed -q 1 -Q 13 -a ‘FORMAT/AD’ -Ou ${bam} | bcftools call -vmO z | bcftools filter -e “%QUAL<20 | ((FMT/AD[0:0]+FMT/AD[0:1])<8 & FMT/AD[0:1]<5)” -s “LowQual” -O u | bcftools filter -e “FMT/AD[0:1]/(FMT/AD[0:0]+FMT/AD[0:1])<0.2” -s “LowFreq” -m + -O u | bcftools sort -O z > ${out}.vcf.gz”

We merged, normalized and left-aligned the mutations that passed the filters using bcftools (HTSlib version 1.7) and we annotated the multi-sample VCFs using ANNOVAR (version 2017-07-17) with the following databases: ensGene (v20170912), 1000g2015aug_all, exac03, avsnp150 and dbnsfp33a. We then filtered out mutations present in any of our 27 normal samples, mutations present in ExAc or 1000 Genomes Project at frequencies above 0.01, mutations that overlapped simple repeats or low complexity regions according to RepeatMasker (downloaded from [http://hgdownload.cse.ucsc.edu/goldenpath/hg38/database/rmsk.txt.gz](http://hgdownload.cse.ucsc.edu/‌goldenpath/hg38/database/rmsk.txt.gz)), and synonymous, intronic, UTR, or upstream/downstream mutations (we did include splice site mutations).

In our second approach, we applied Mutect2 (GATK version 4.1.4.0) in non-paired mode using default parameters, an in-house PoN and gnomAD v3 as a germline resource. The in-house PoN was constructed using data from 27 normal adjacent tissue samples from LUAD patients, including any mutation found in at least one normal sample (`--min-sample-count 1` option in `CreateSomaticPanelOfNormals`). We compared our two approaches for the non-paired analyses and we individually evaluated the discrepancies between the two pipelines using Integrative Genomics Viewer (v2.3.94) and public databases (see Supplementary Note). We decided to combine the results from the BCFtools analysis with manually ‘rescued’ mutations from the Mutect2 approach after careful inspection of the Mutect2-exclusive mutations. We flagged such mutations as ‘Mutect2’.

2. Supplemental Note

CCLE Comparison with Our Mutation Data

Thirty-one of our 38 cell lines had also been sequenced by CCLE. Within these cell lines, 46 alterations were found in the 20 SWI/SNF subunits in the CCLE database in contrast with the 44 mutations identified in our study. In general, the results of our analysis were highly concordant with the mutations of CCLE. However, we detected and confirmed by Sanger sequencing three mutations that were not previously reported in CCLE: a splice site mutation in *SMARCA4* in H2122, which affects the isoform NM_001128845, and two non-synonymous mutations in PHF10 and BRD9 in H1734 and H1975, respectively (see Figure S2).

On the other hand, in our analysis we did not find 9 mutations described in CCLE, after accounting for differences in the annotation reference (Figure S3). Four of these mutations were inframe indels or missense mutations that were filtered out in our analysis because they affected low complexity regions of ARID1B (Calu-3 and H1648), ARID1A (H441), or SMARCC2 (H1623) (Figure S4). In addition, we did not find 5 non-synonymous mutations in PBRM1 (PC14 and H1975), SMARCC1 (A549), SMARCD2 (H2122), and DPF3 (H23). In the last case, we detected the mutation but it did not pass our filtering criteria due to low sequencing depth (Figure S5).

3. Supplemental Figures


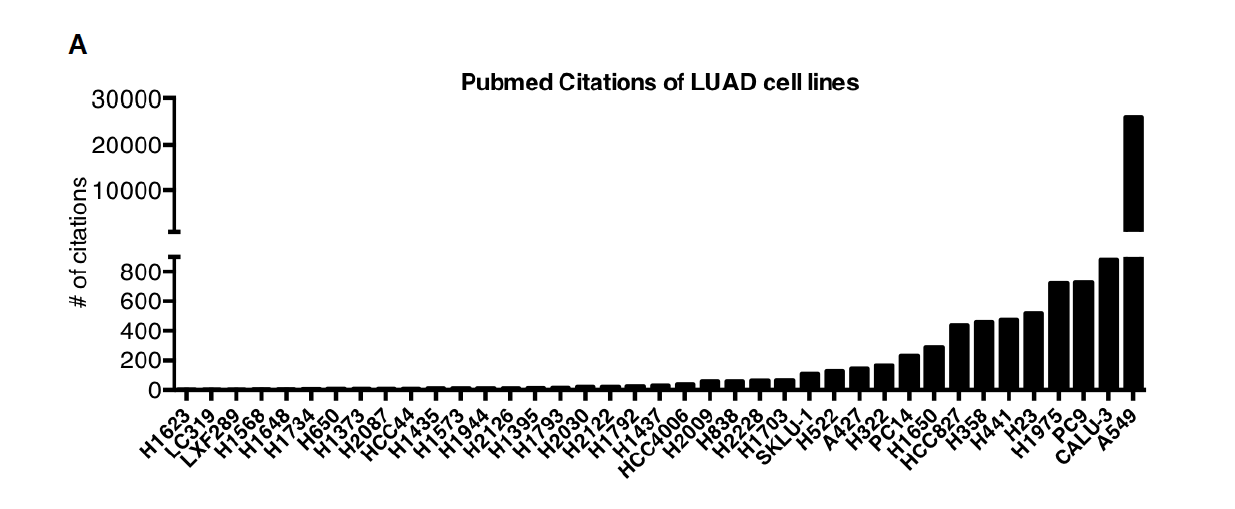


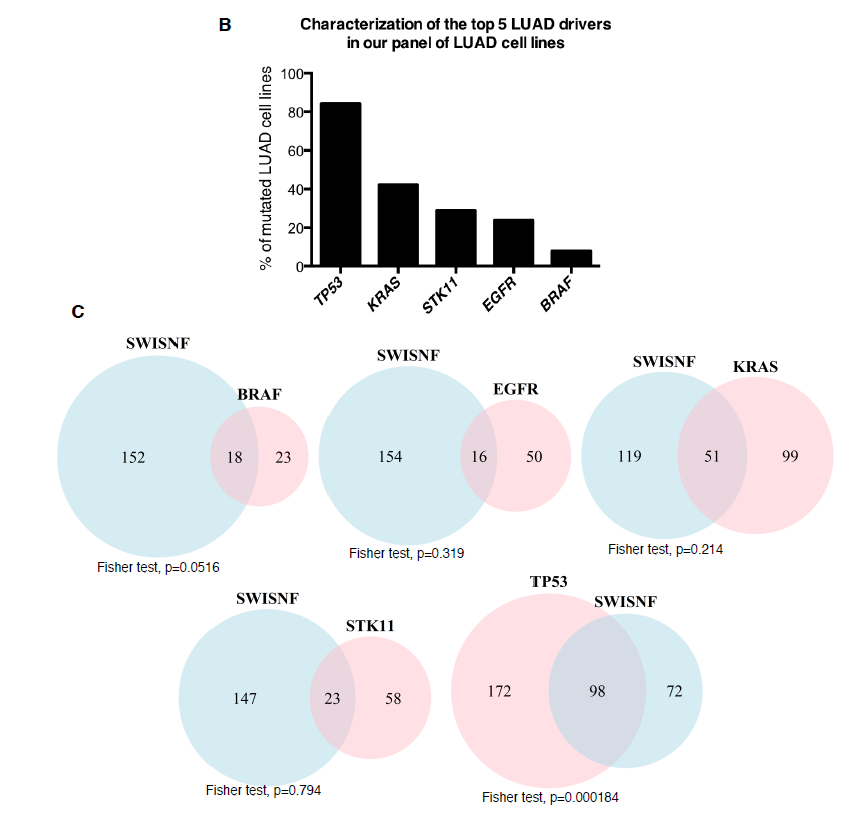


**Figure S1.** (**A**) Number of citations of each of the LUAD cell lines used in this study according to PubMed until September 2019. (**B**) Mutation profile of the top 5 LUAD driver genes identified by Bailey and colleagues in our panel of 38 LUAD cell lines. (**C**) Venn Diagrams with the mutational status of the top 5 LUAD driver genes and the SWI/SNF complex in LUAD patients using TCGA-LUAD data.


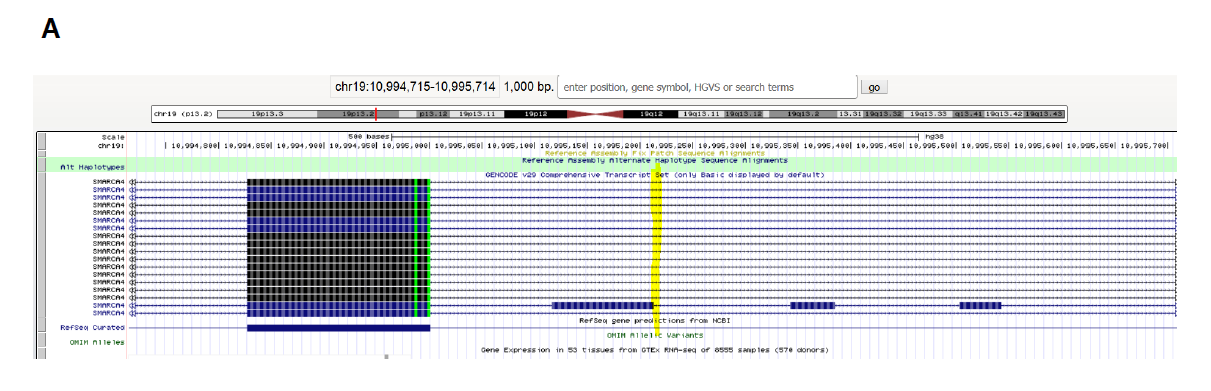


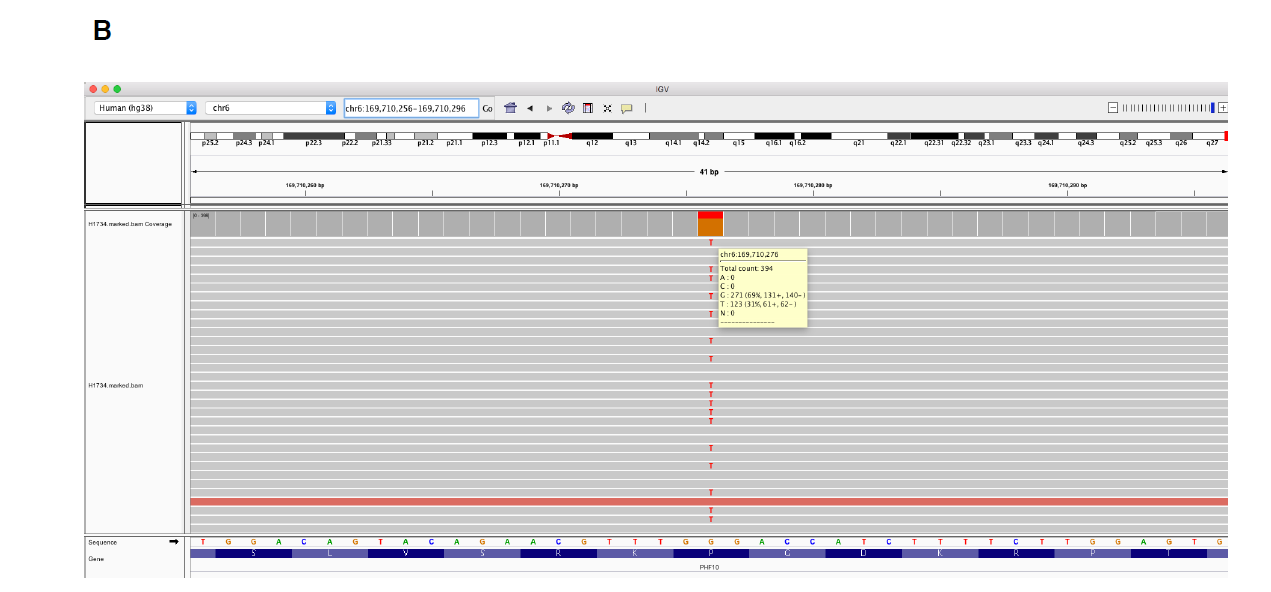


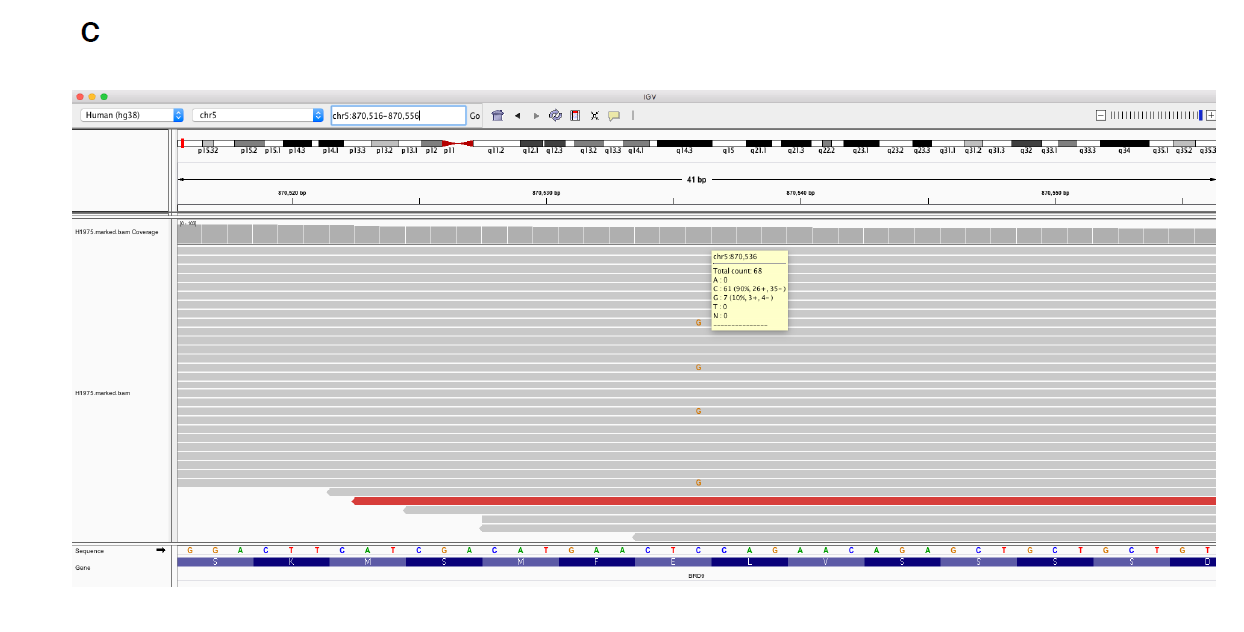


**Figure S2.** De novo SWI/SNF complex mutations identified by our study in LUAD cell lines. (**A**) UCSC genome browser screenshot of the SMARCA4 isoform that is mutated in the H2122 cell line (NM_001128845) according to our sequencing data. (**B**) PHF10 mutation in the H1734 cell line (chr6:169710276 G-T). (**C**) BRD9 mutation in the H1975 cell line (chr5:870536 C>G). All genomic coordinates are given with hg38 annotation.


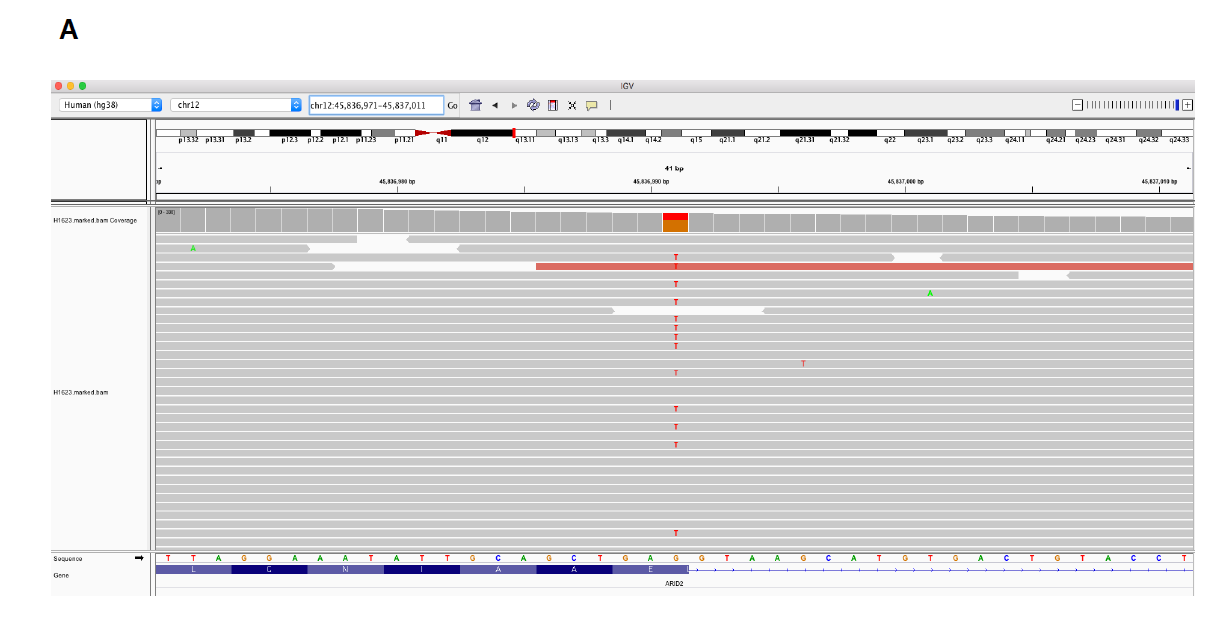


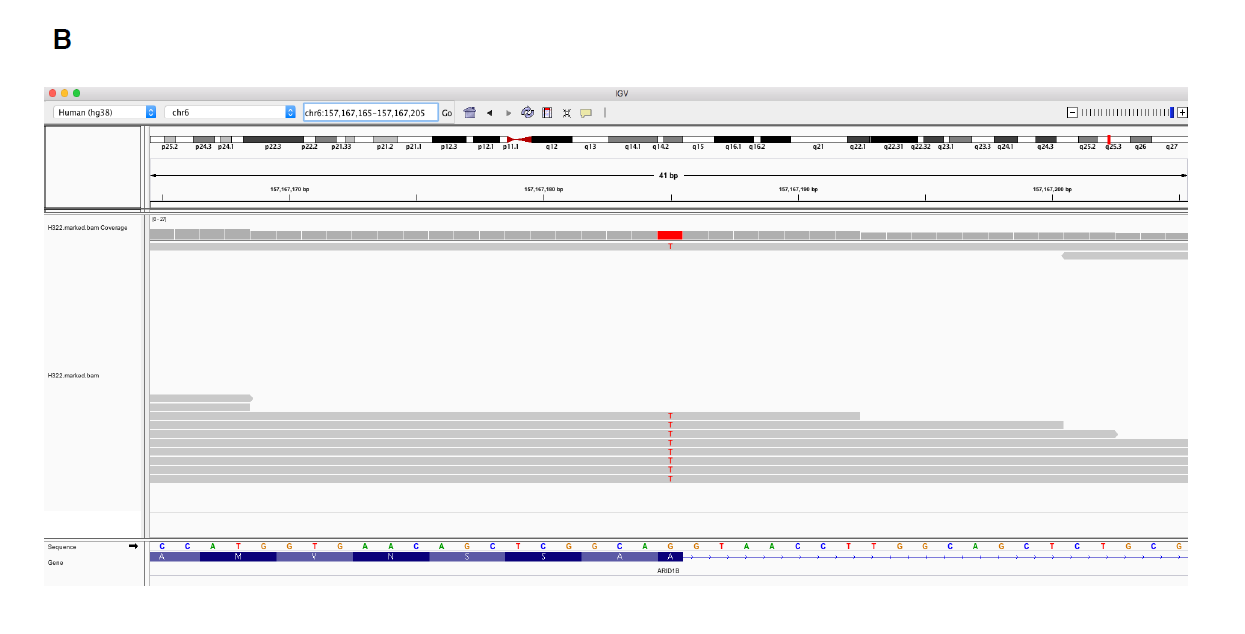


**Figure S3.** SWI/SNF complex mutations with difference in annotation between CCLE and our data. (**A**) ARID2 mutation in the H1623 cell line (chr12:45836991 G>T). (**B**) ARID1B mutation in the H322 cell line (chr6:157167185 G>T). All genomic coordinates are given with hg38 annotation.


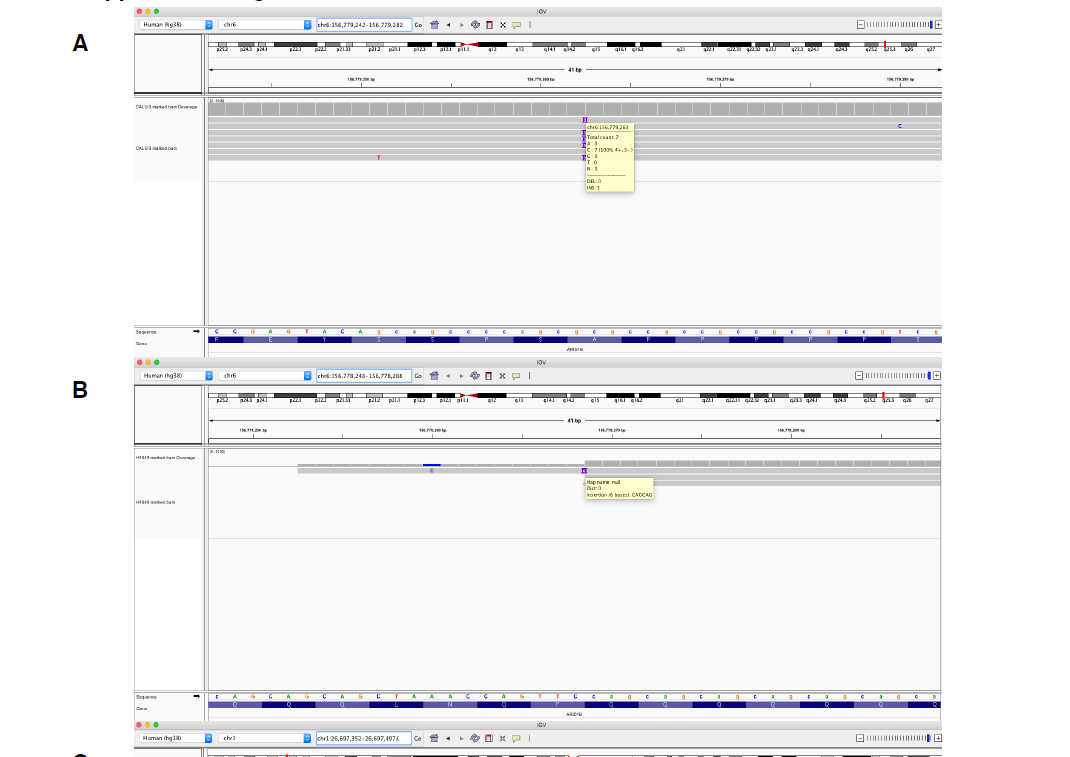


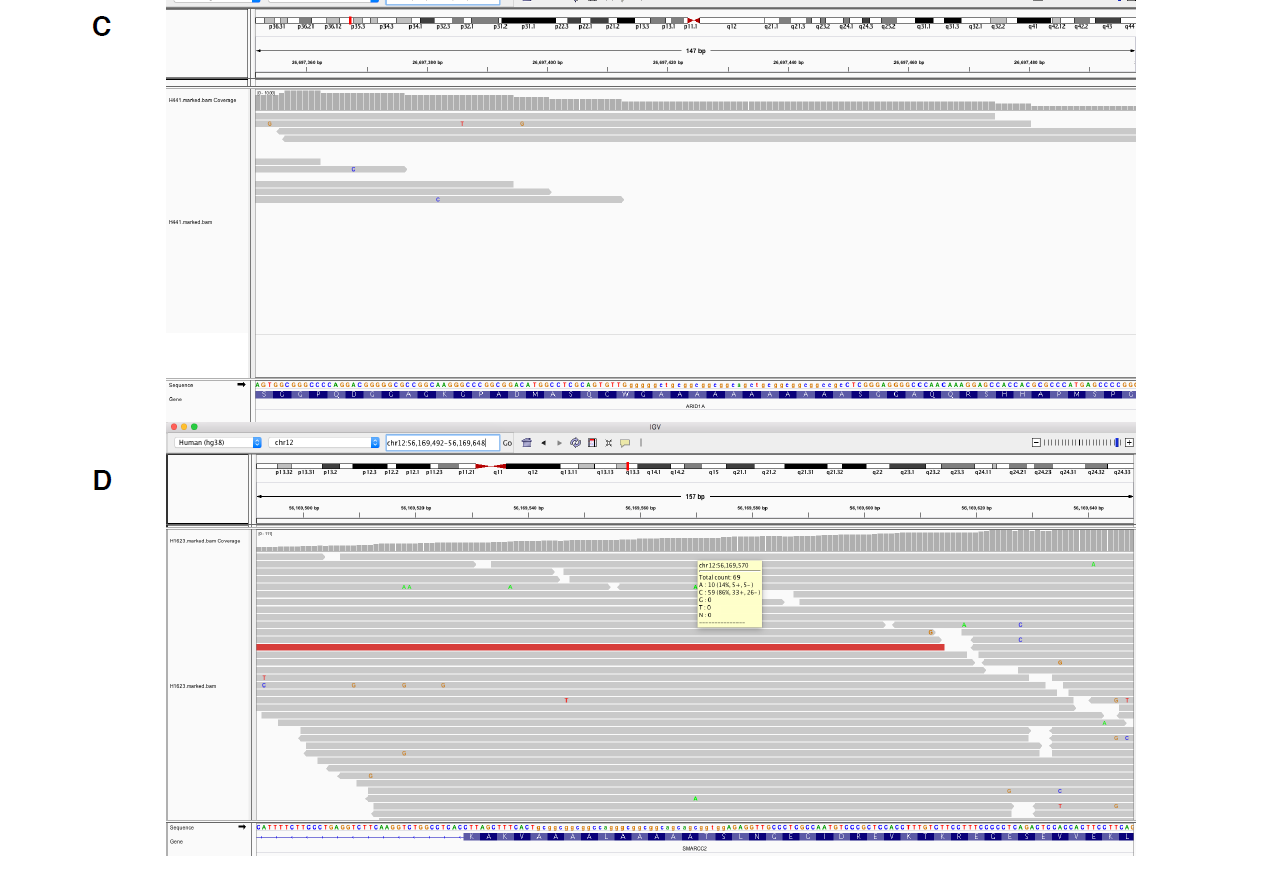


**Figure S4.** CCLE mutations in the lung SWI/SNF complex excluded in our analysis because of affecting low complexity genomic regions (**A**) ARID1B insertion in the Calu-3 cell line (chr6:156779262-156779263 ins CGC). (**B**) ARID1B insertion in the H1648 cell line (chr6:156778268-156778269 ins CAGCAG). (**C**) ARID1A deletion in the H441 cell line (chr1: 26697418-26697432 inframe del). (**D**) SMARCC2 mutation in the H1623 cell line (chr12:56169570 C>A). All genomic coordinates are given with hg38 annotation.


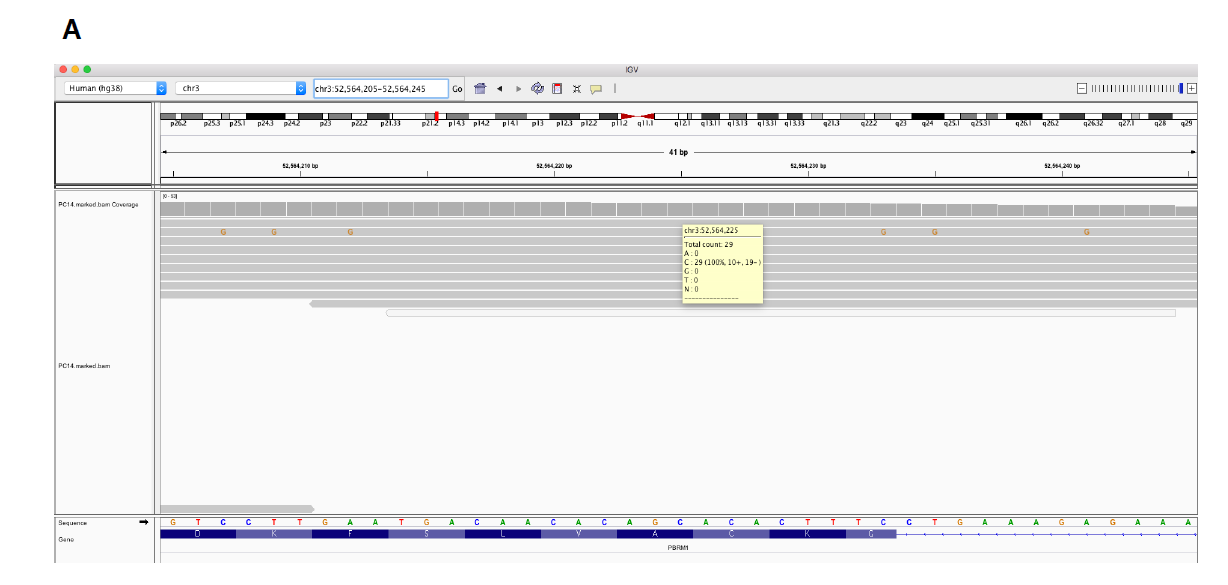


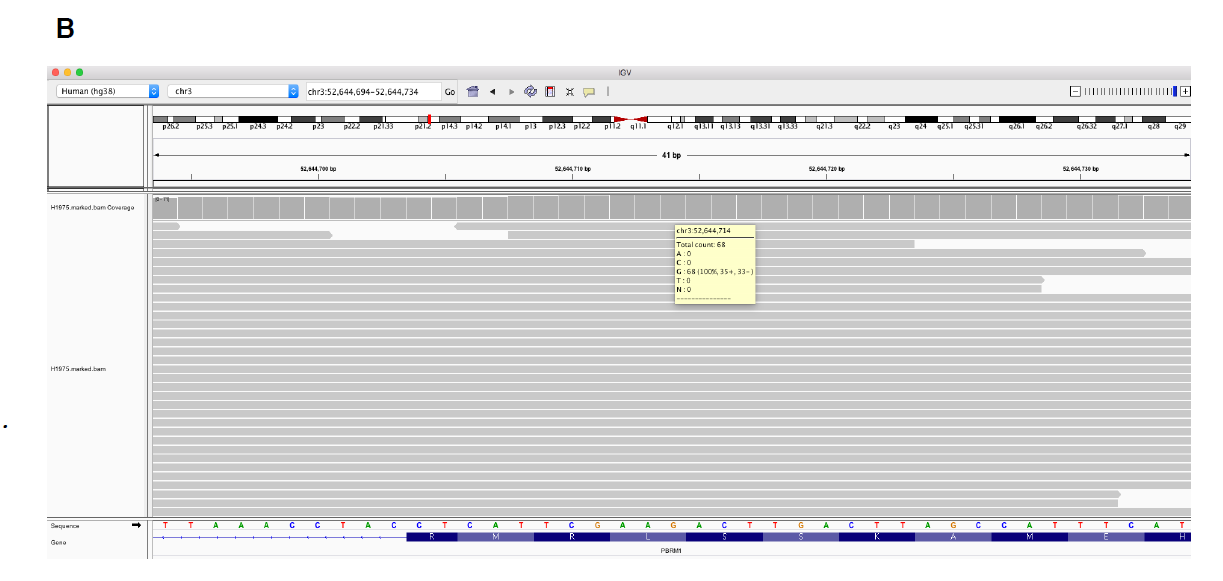


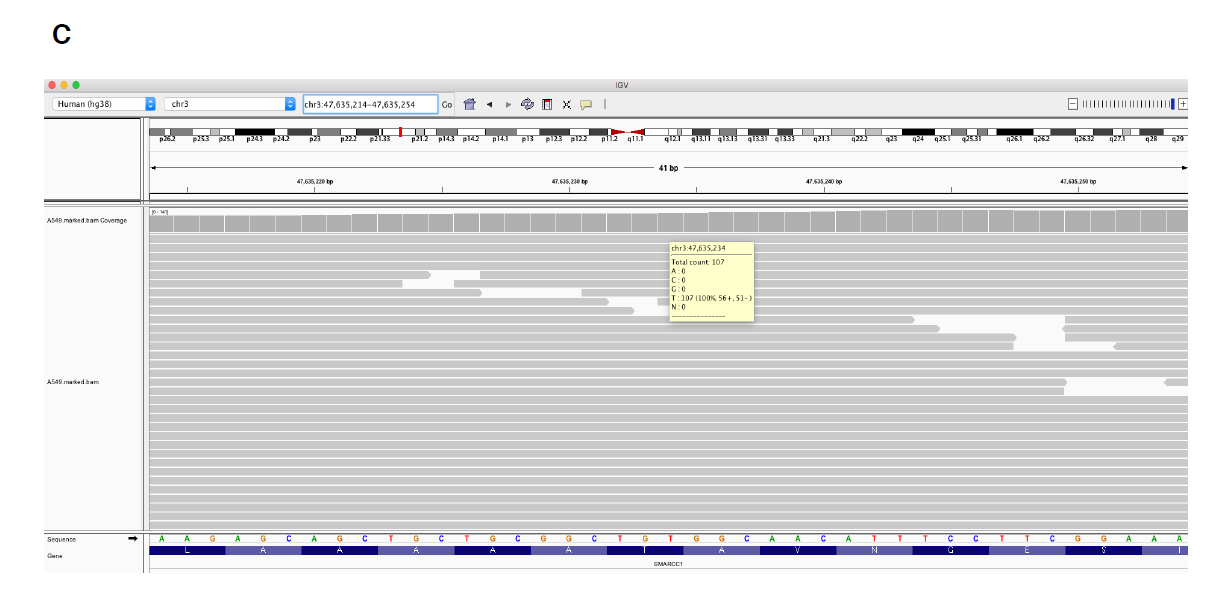


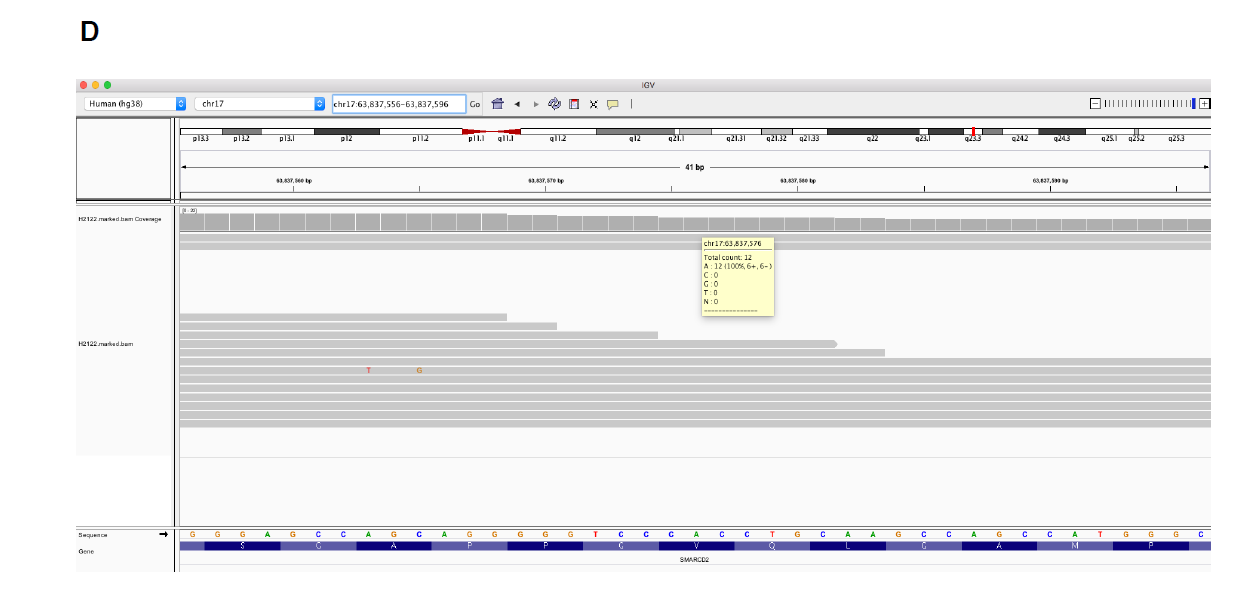


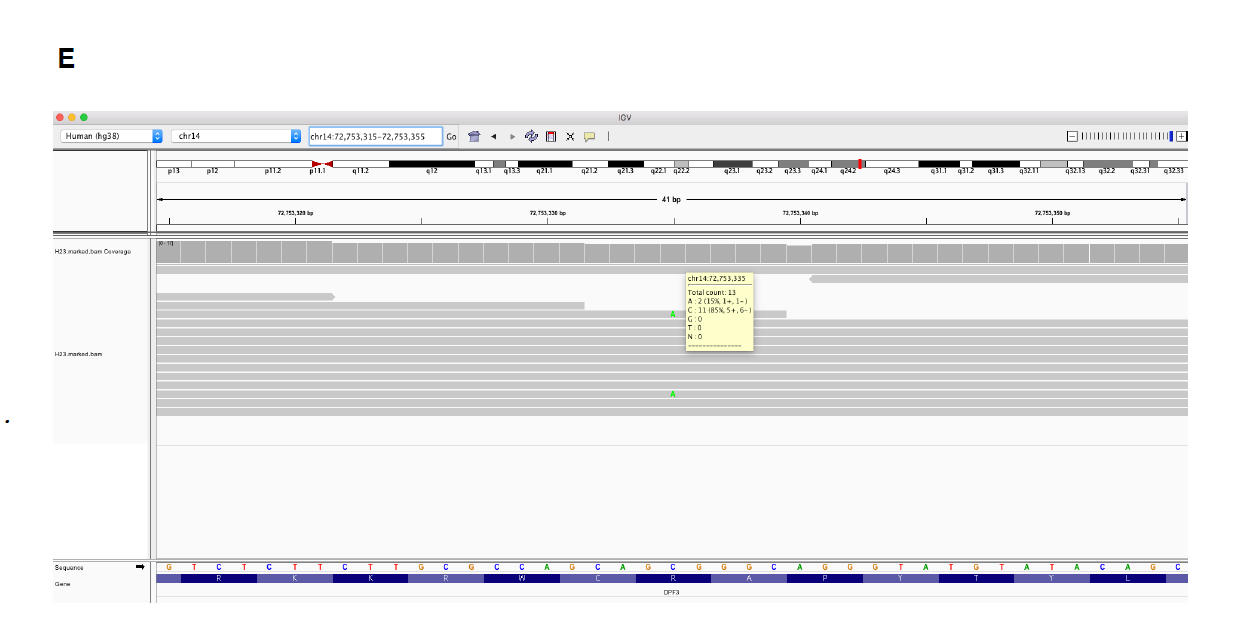


**Figure S5.** Lung SWI/SNF complex mutations exclusively described in CCLE. (**A**) PBRM1 mutation in the PC14 cell line (chr3:52564225 C>T). (**B**) PBRM1 mutation in the H1975 cell line (chr3:52644714 G>T). (**C**) SMARCC1 mutation in the A549 cell line (chr3:47635234 T>G). (**D**) SMARCD2 mutation in the H2122 cell line (chr17:63837576 A>C). (**E**) DPF3 mutation in the H23 cell line (chr14:72753335 C>A). All genomic coordinates are given with hg38 annotation.


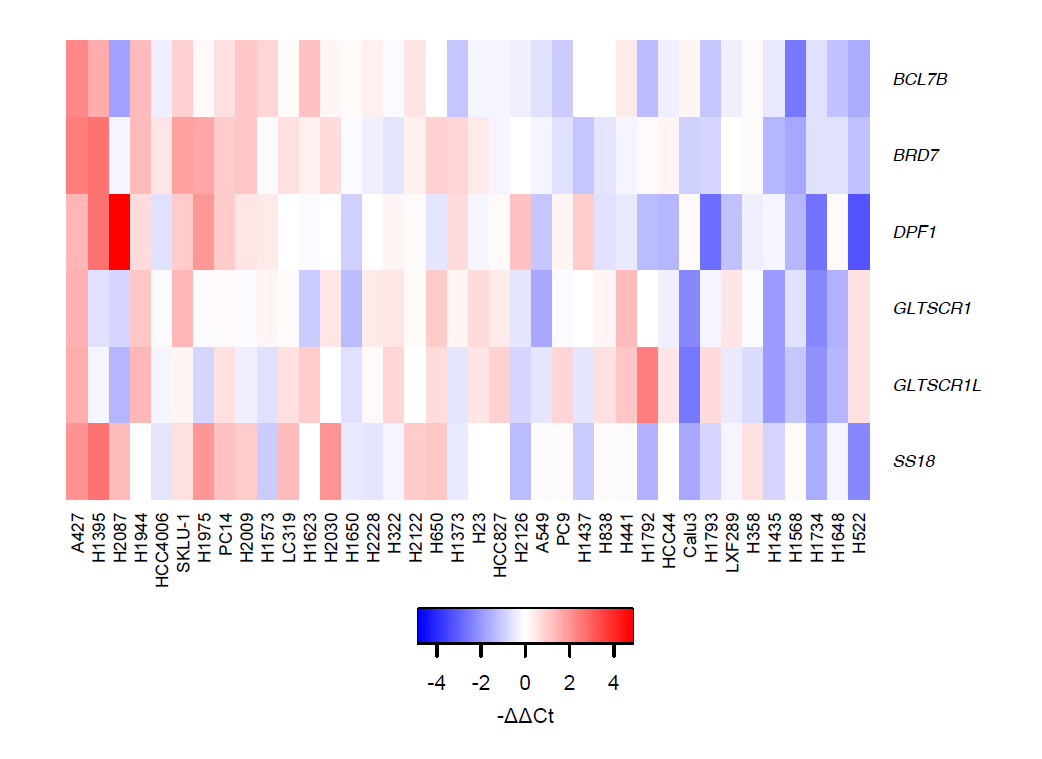


**Figure S6.** Heatmap of mRNA expression changes of additional SWI/SNF subunits in our 38 LUAD cell lines (-DDCt was calculated using the median DCt for each of the measured genes). *Y* axis represents all measured SWI/SNF subunits. *X* axis contains the 38 LUAD cell lines of our study.


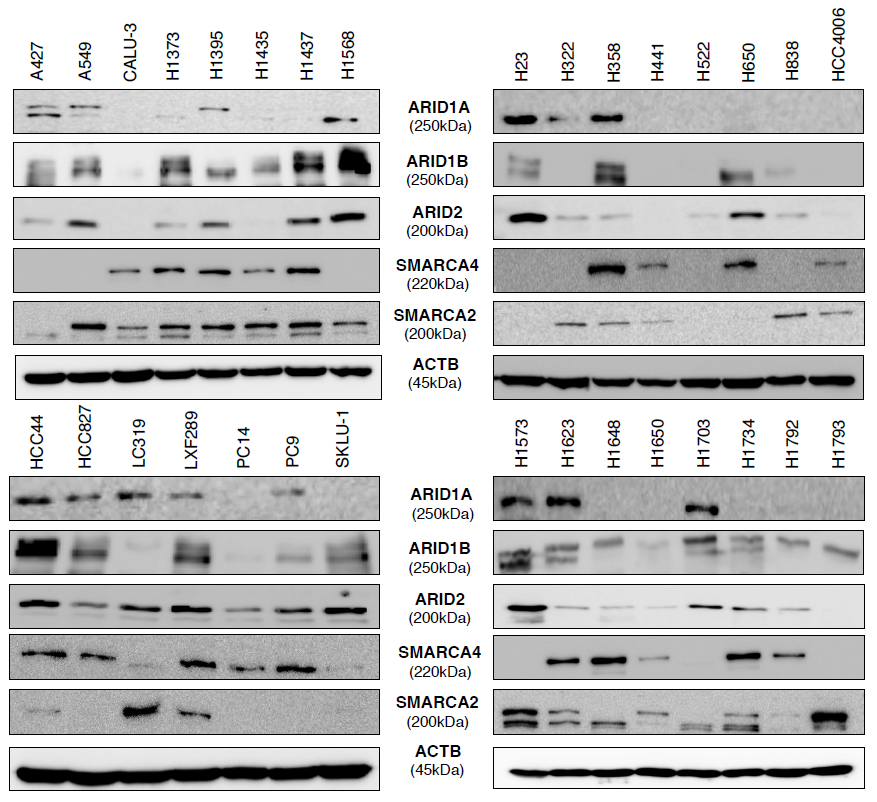


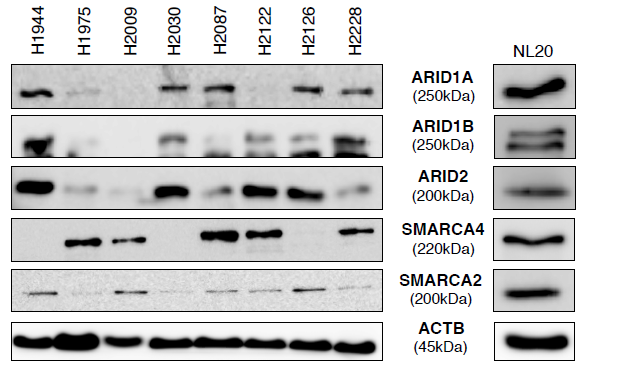


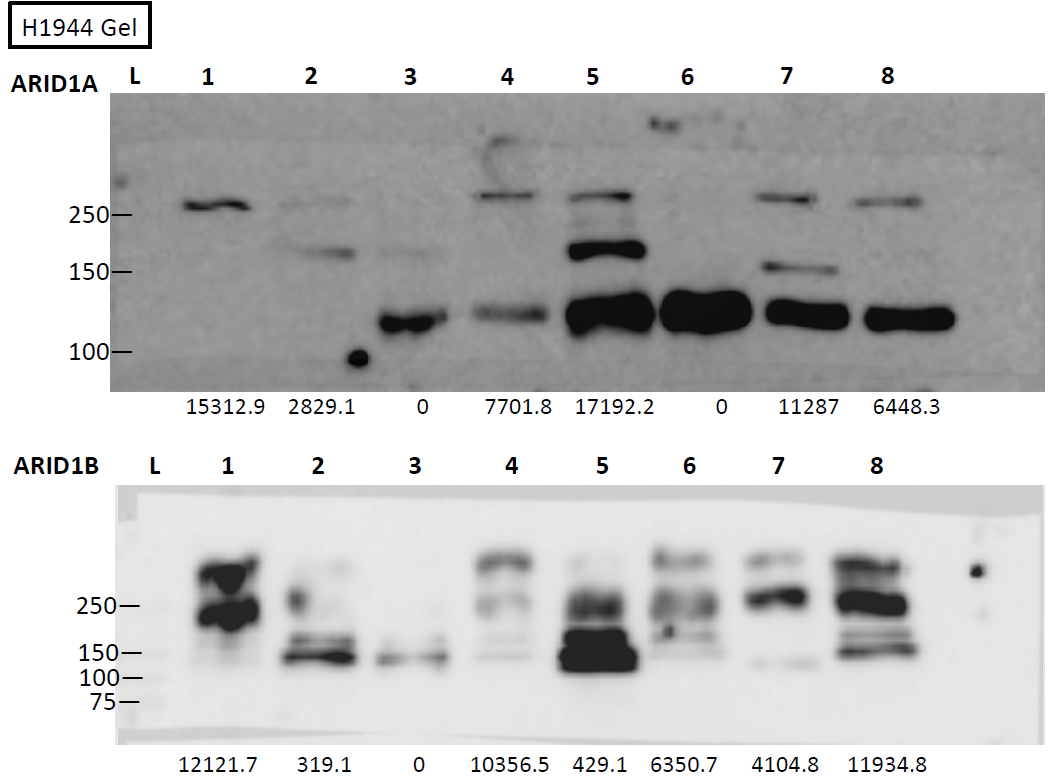


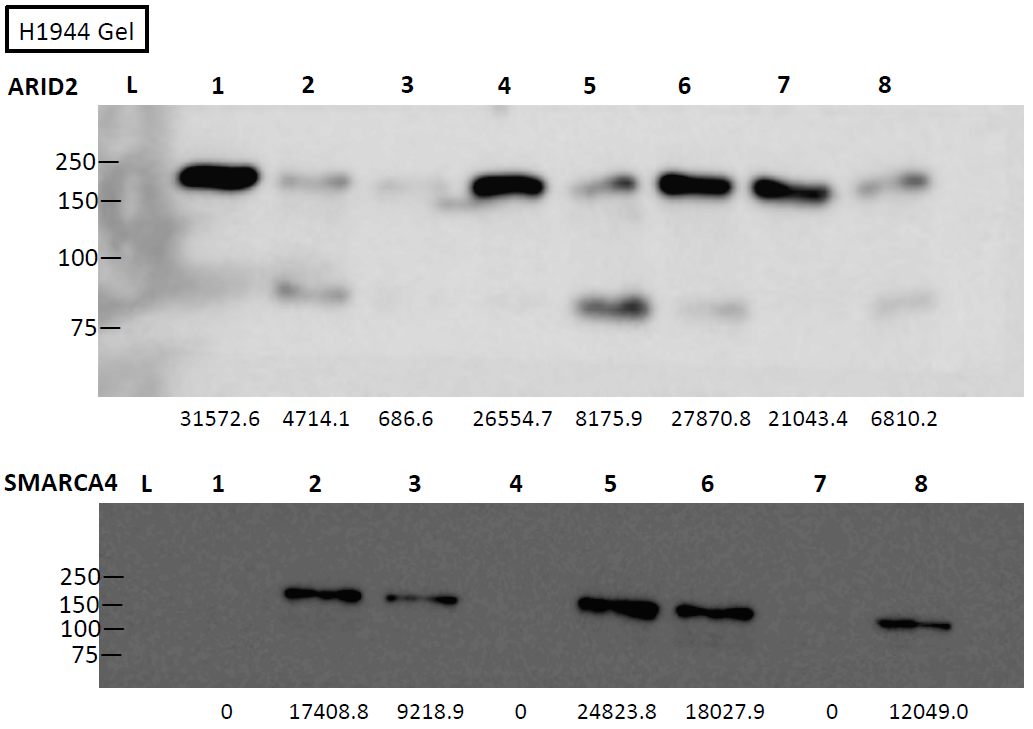


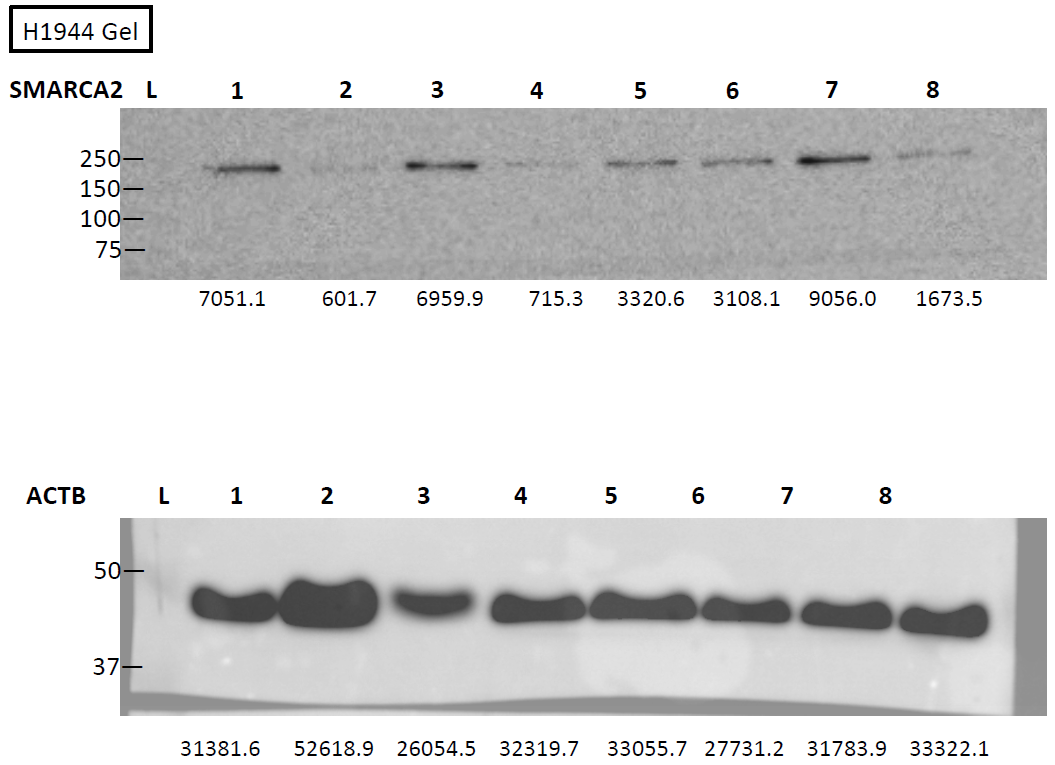


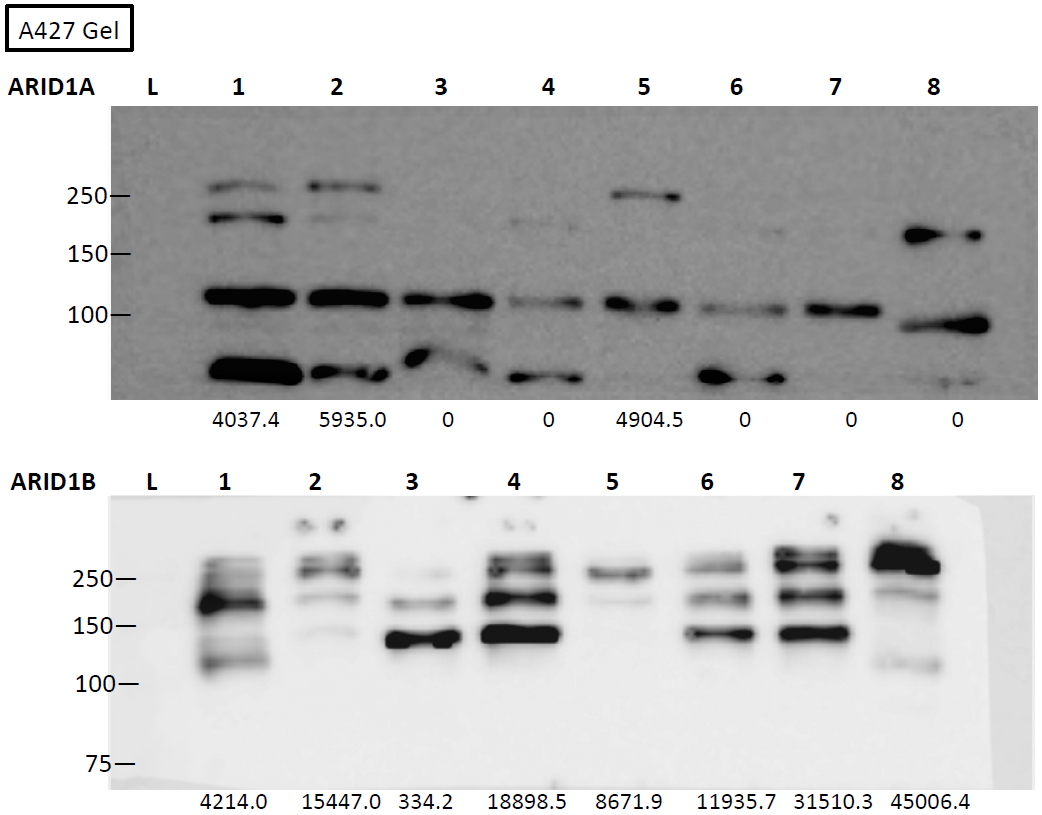


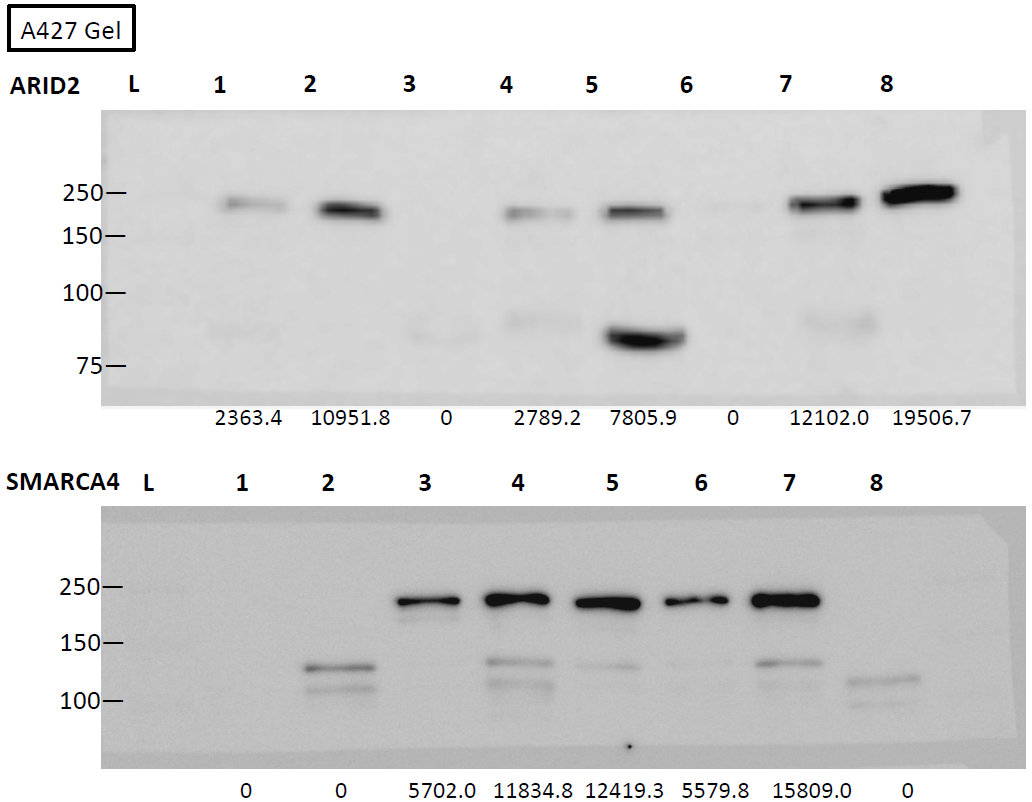


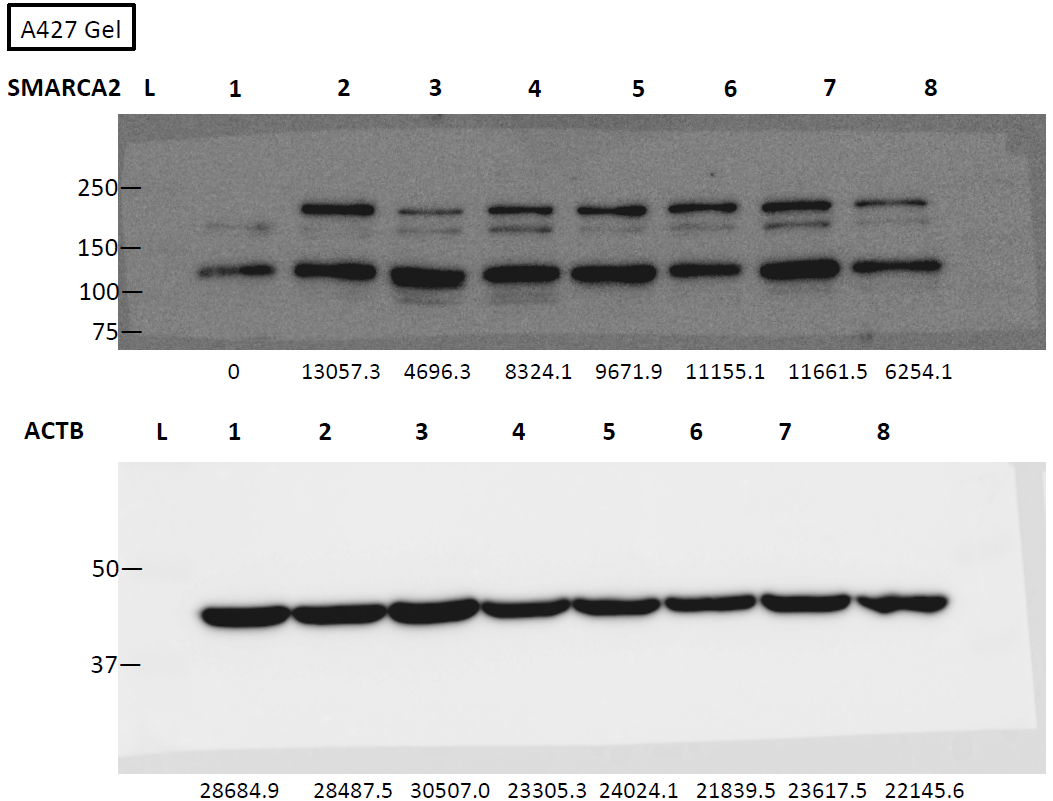


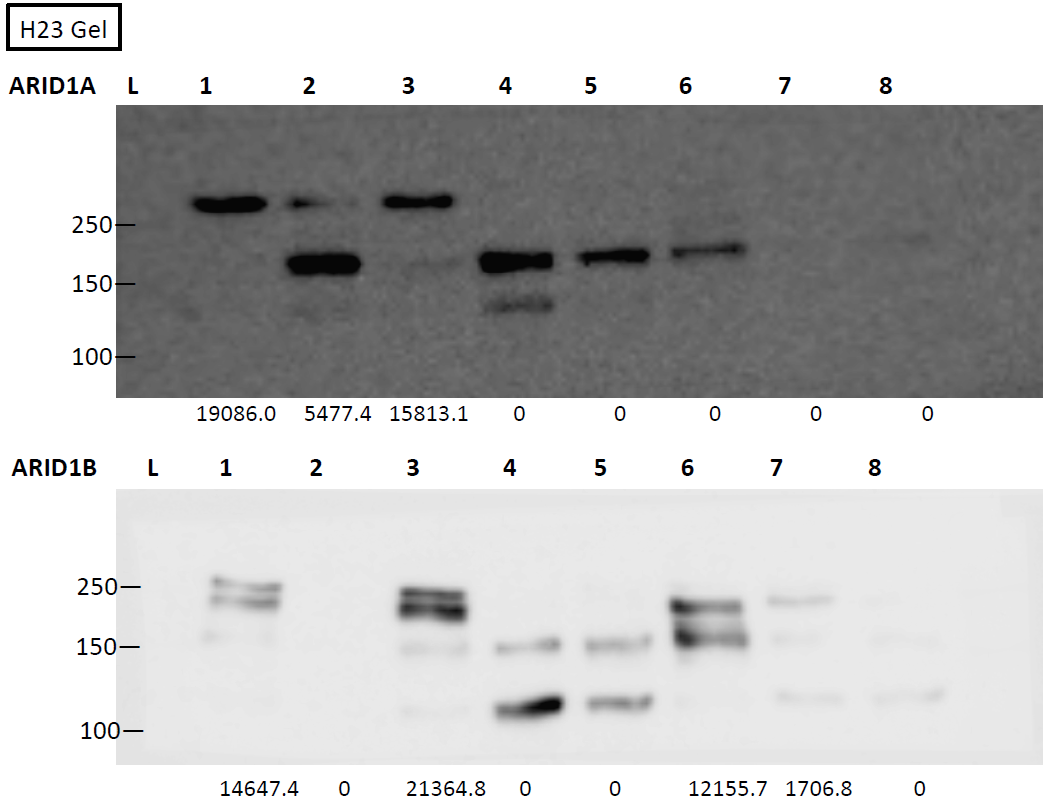


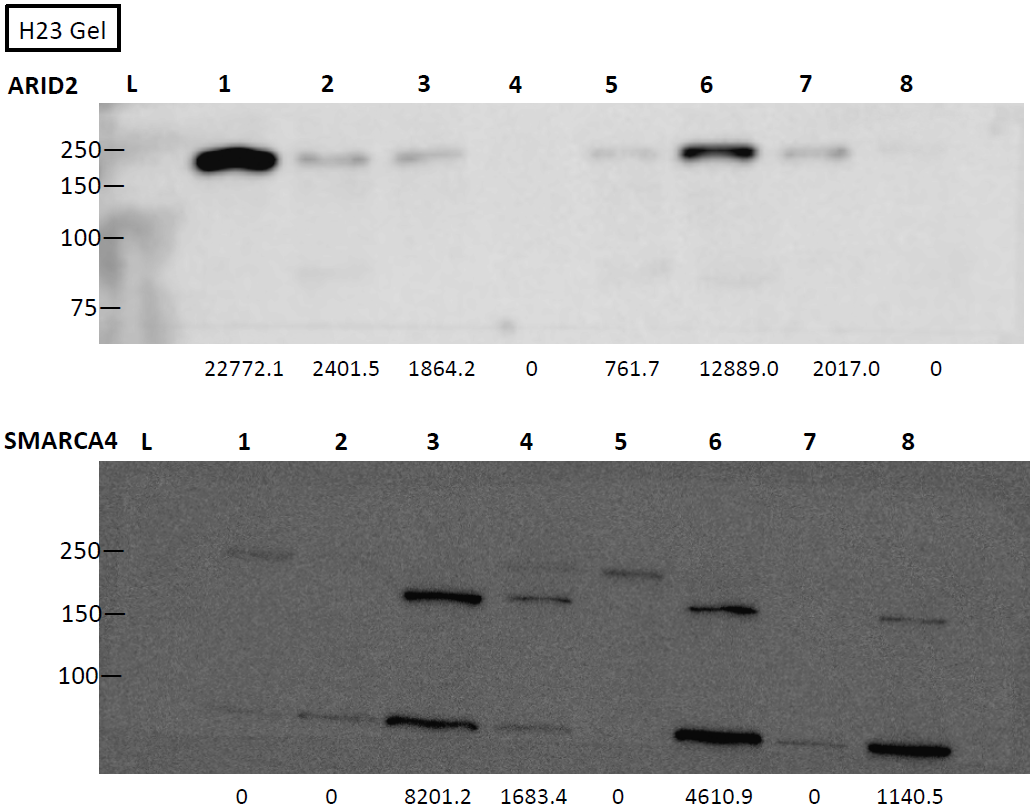


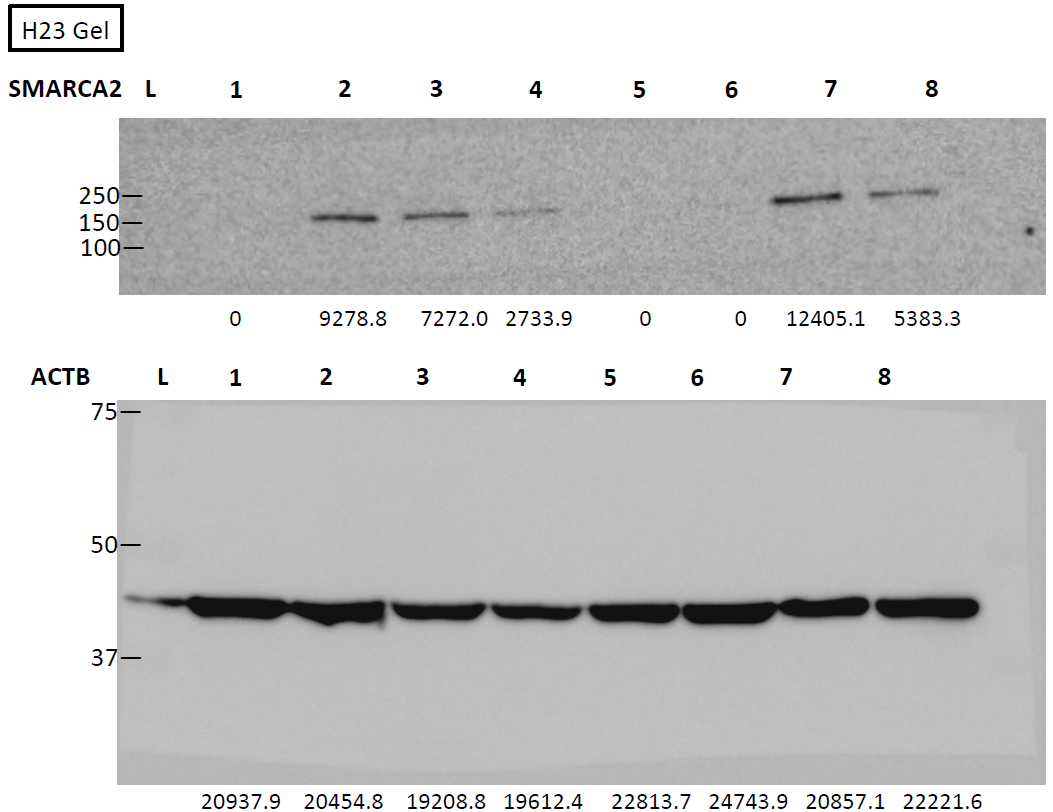


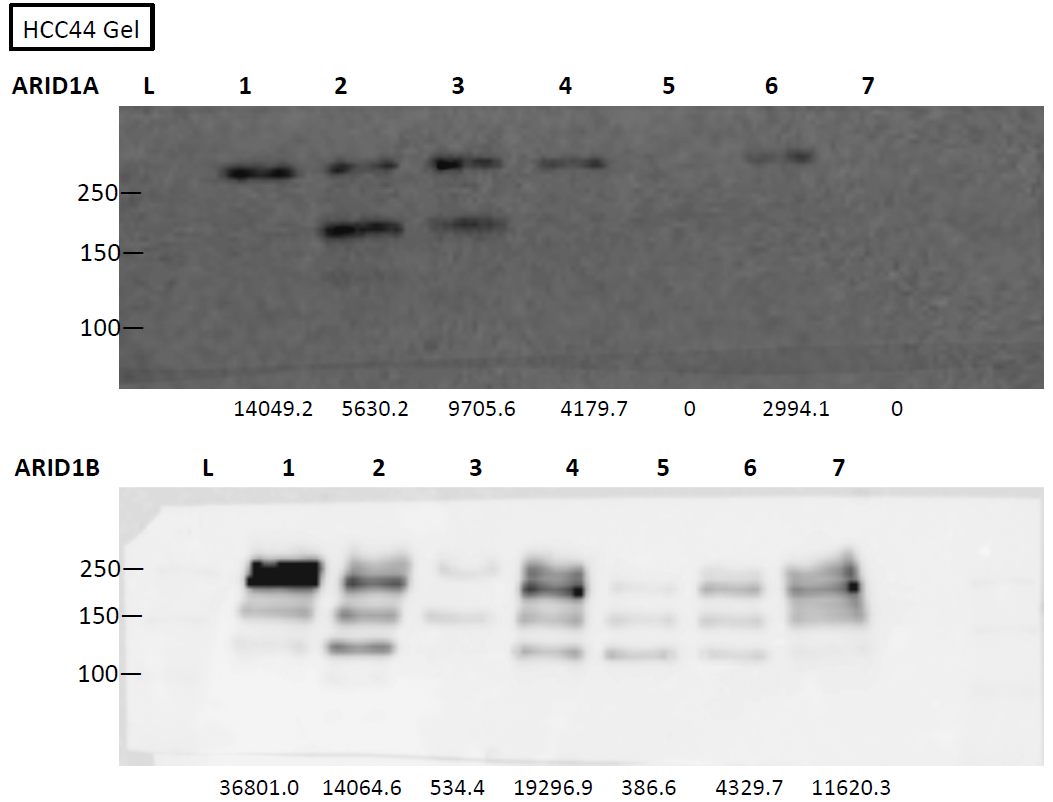


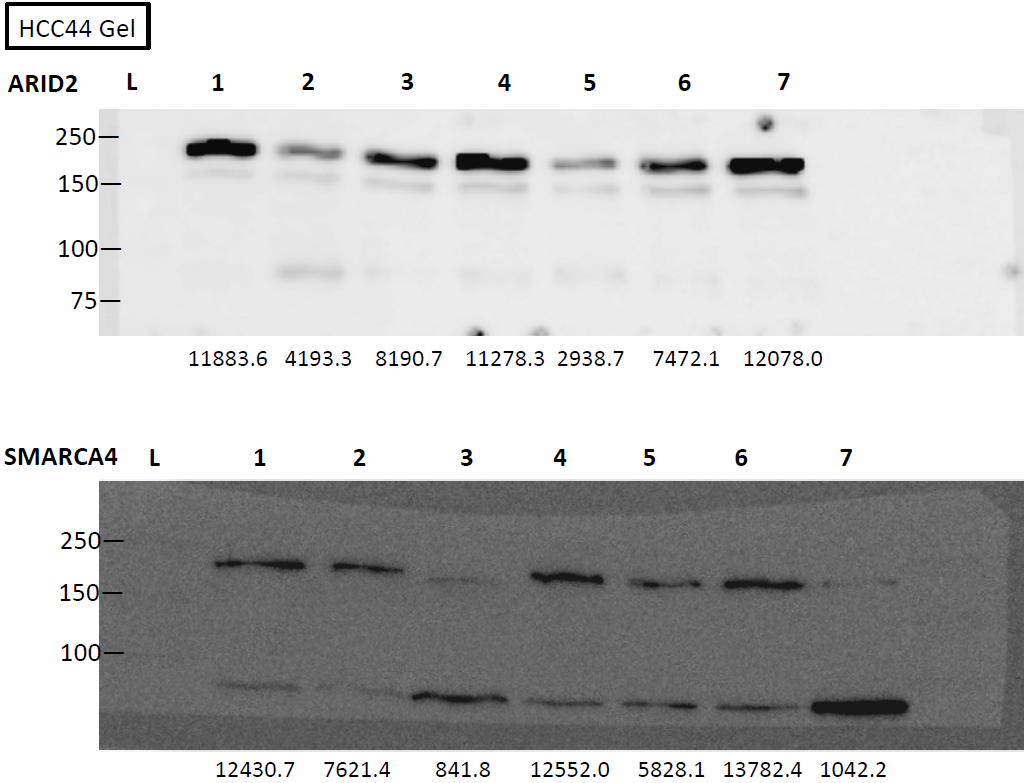


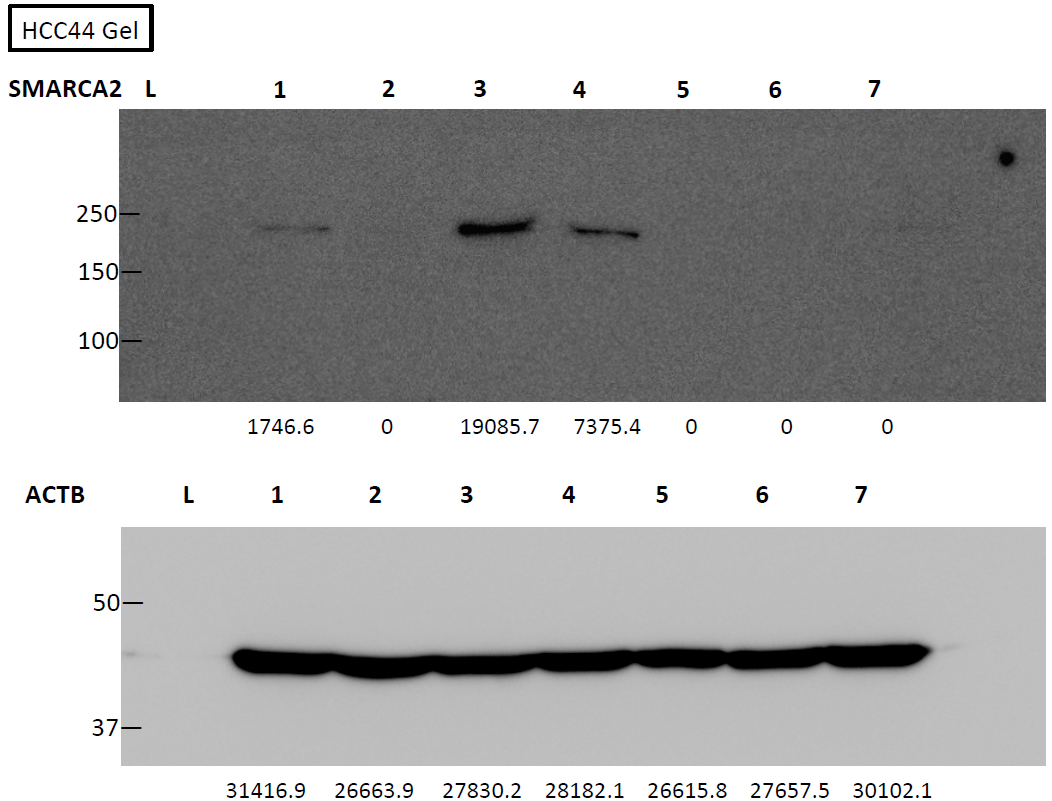


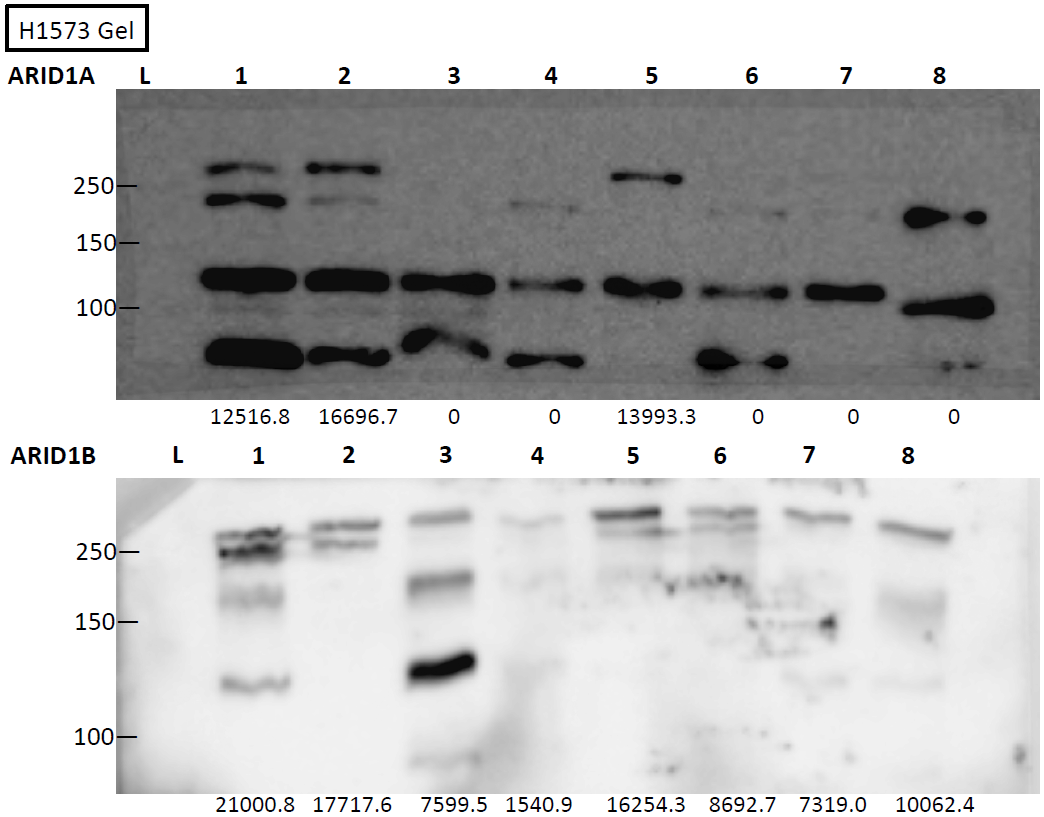


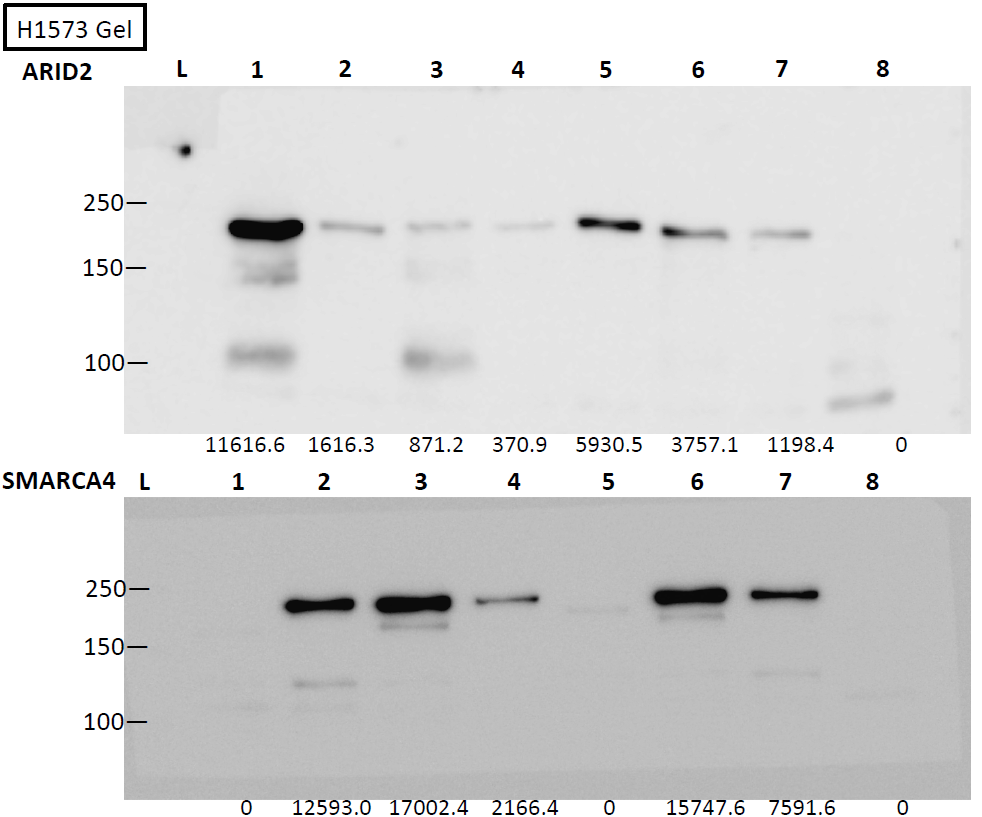


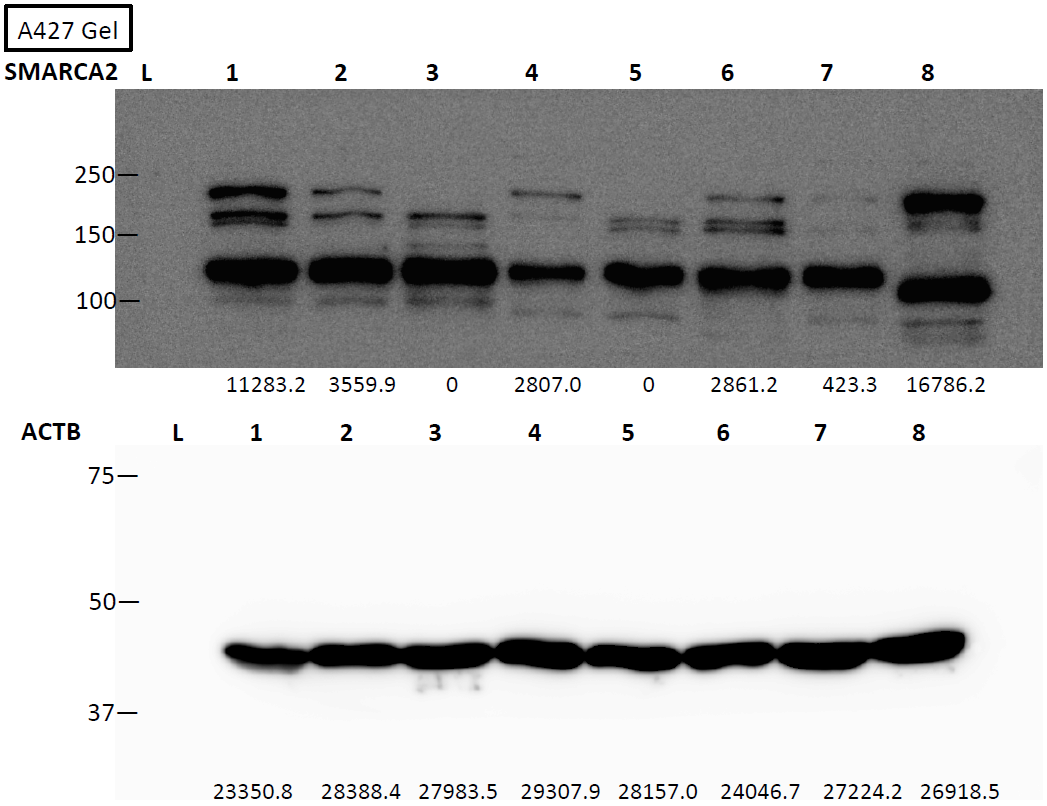


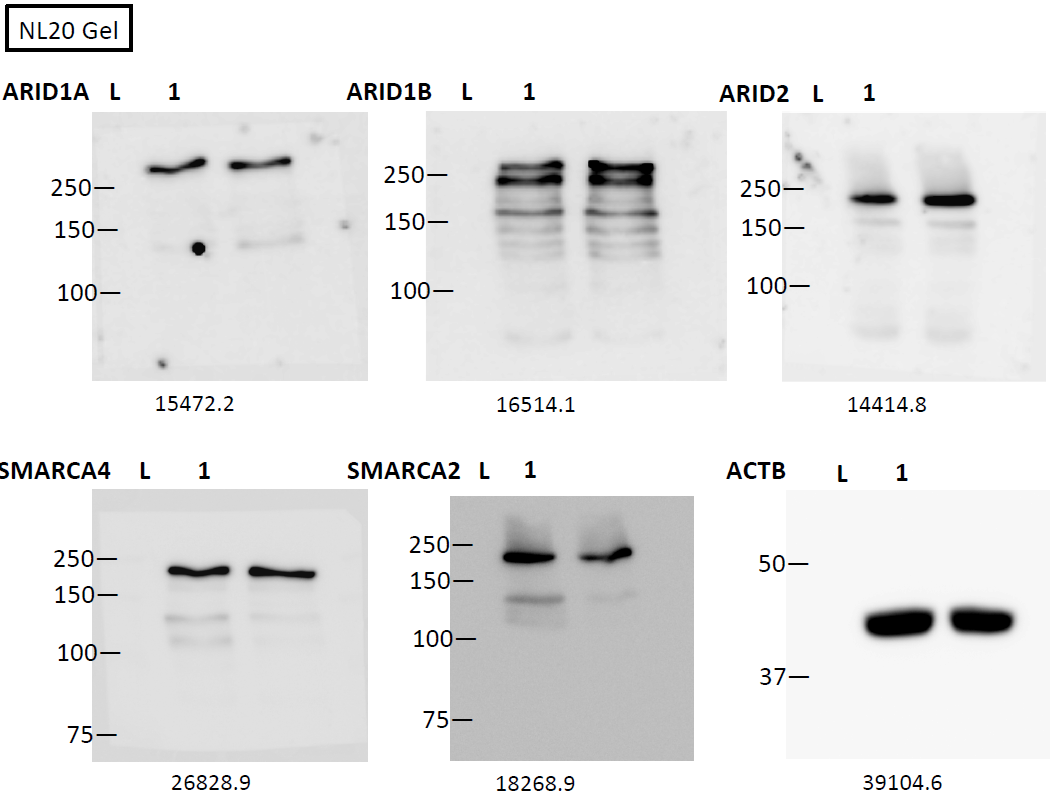


**Figure S7.** Western blot of SMARCA4, SMARCA2, ARID1A, ARID1B, and ARID2 in our 38 LUAD cell lines and a normal lung cell line (NL20). The sizes depicted in the image correspond to the observed molecular weights of each of these proteins.


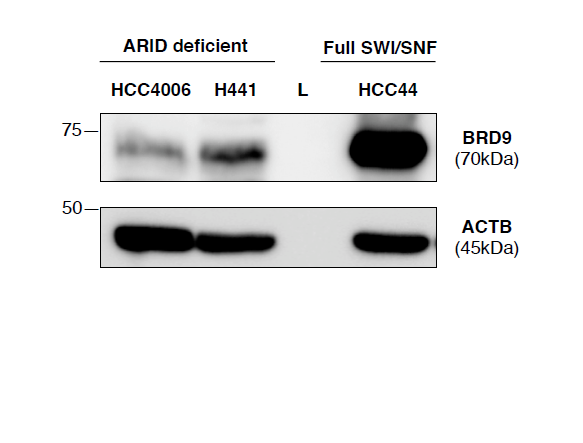


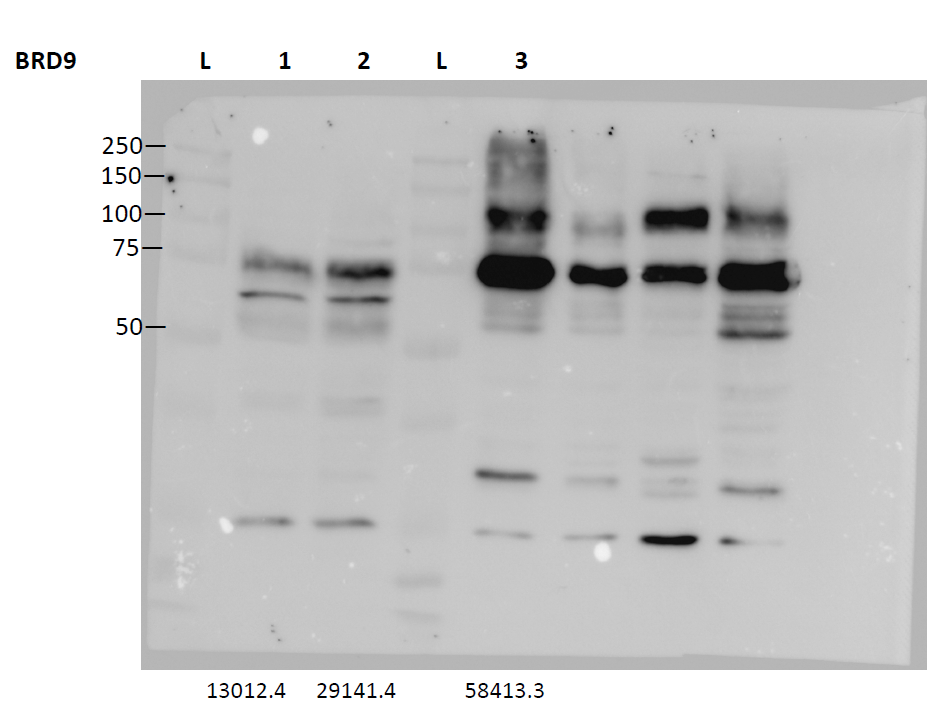


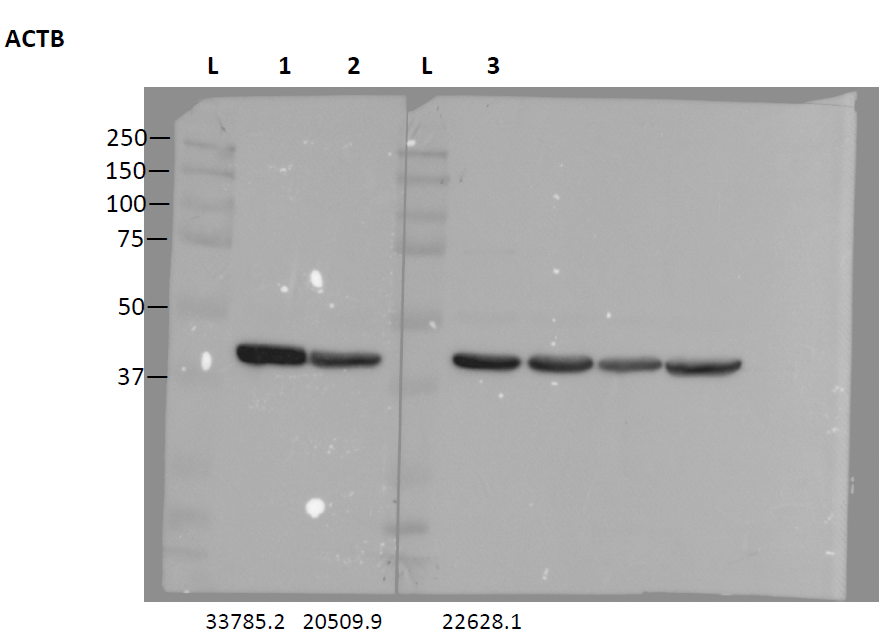


**Figure S8.** Western blot of BRD9 in our ARID deficient LUAD cell lines and a FULL-SWI/SNF LUAD cell line. The sizes depicted in the image correspond to the observed molecular weight of BRD9.

4. Supplemental References

1. Martin, M. Cutadapt removes adapter sequences from high-throughput sequencing reads. *EMBnet J.* **2011**, *17*, 3–12.
2. García-Alcalde, F.; Okonechnikov, K.; Carbonell, J.; Cruz, L.M.; Götz, S.; Tarazona, S.; Dopazo, J.; Meyer, T.F.; Conesa, A. Qualimap: evaluating next-generation sequencing alignment data. *Bioinformatics* **2012***, 28*, 2678–2679.
3. Ewels, P.; Magnusson, M.; Lundin, S.; Käller, M. MultiQC: summarize analysis results for multiple tools and samples in a single report. *Bioinformatics* **2016***, 32*, 3047–3048.

**Publisher’s Note:** MDPI stays neutral with regard to jurisdictional claims in published maps and institutional affiliations.

| 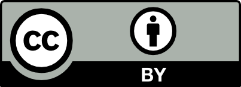 | © 2020 by the authors. Licensee MDPI, Basel, Switzerland. This article is an open access article distributed under the terms and conditions of the Creative Commons Attribution (CC BY) license (http://creativecommons.org/licenses/by/4.0/). |
| --- | --- |
